# RNA delivery to the corneal endothelium using charge-altering releasable transporters

**DOI:** 10.1126/sciadv.ady8161

**Published:** 2026-08-21

**Authors:** Sean K. Wang, Zhijian Li, Sahil H. Shah, Quincy A. Edwards, Richard Bouffard, Elizabeth S. Hines, Joel A. Imventarza, Sven Korte, Matthew S. Lawrence, Euisun Song, Ekram Helmy, Laura Amaya, Nae-Won Kang, David Myung, Miao-Chih Tsai, William J. Greenleaf, Robert M. Waymouth, Sui Wang, Paul A. Wender, Howard Y. Chang

**Affiliations:** ^1^ Center for Personal Dynamic Regulomes, Stanford University, Stanford, CA, USA.; ^2^ Department of Ophthalmology, Stanford University School of Medicine, Stanford, CA, USA.; ^3^ Department of Chemistry, Stanford University, Stanford, CA, USA.; ^4^ Virscio, Inc., New Haven, CT, USA.; ^5^ RNA Medicine Program, Stanford University, Stanford, CA, USA.; ^6^ College of Pharmacy, The Catholic University of Korea, Bucheon, Republic of Korea.; ^7^ Department of Chemical Engineering, Stanford University, Stanford, CA, USA.; ^8^ VA Palo Alto Health Care System, Palo Alto, CA, USA.; ^9^ Department of Genetics, Stanford University, Stanford, CA, USA.; ^10^ Department of Chemical and Systems Biology, Stanford University, Stanford, CA, USA.; ^11^ Department of Dermatology, Stanford University School of Medicine, Stanford, CA, USA.

## Abstract

RNA therapies hold tremendous promise for treating genetic eye diseases. However, their development is limited by the lack of non-viral delivery platforms that can target specific ocular cell types. Here, we describe a charge-altering releasable transporter (CART) that delivers RNA selectively to the corneal endothelium, a non-regenerative cell layer whose dysfunction underlies several blinding conditions. We characterize the safety of CART-RNA nanoparticles in mice and show that they facilitate delivery of diverse RNA cargoes to the corneal endothelium, including circular RNA and CRISPR/Cas9. We verify that these nanoparticles can be redosed and apply them to achieve corneal gene editing. We further demonstrate CART transfection of corneal endothelial cells from a human donor in vitro and in a non-human primate in vivo, supporting the feasibility of clinical translation. Our findings establish CARTs as a platform for non-viral gene delivery to the eye, with the potential to treat corneal dystrophies and other vision disorders.

## INTRODUCTION

RNA medicines have emerged as a promising therapeutic class to address a broad spectrum of pathologies, ranging from rare genetic disorders to widespread infectious diseases (*1*, *2*). Paralleling the rise of RNA therapies have been advances in non-viral nucleic acid delivery platforms, such as lipid nanoparticles (LNPs) and polymeric nanocarriers (*3*–*8*). In contrast to viral vectors, these nanoparticle-based systems mediate transient gene expression, offering control over the timing of gene activity while minimizing the risk of genomic integration or immune activation (*9*–*11*). Together, these technologies made possible the development of mRNA vaccines against coronavirus disease 2019 (COVID-19) (*12*, *13*) and are now being adapted for protein replacement therapies (*14*, *15*), gene editing approaches (*16*, *17*), and personalized cancer treatments (*18*, *19*).

The eye is an attractive target for RNA medicines due to its accessible anatomy, small size, compartmentalized structure, and relative immune tolerance (*20*, *21*). Indeed, pegaptanib (Macugen), an RNA aptamer injected into the vitreous of the eye to treat neovascular age-related macular degeneration (*22*), was among the first RNA-based drugs approved by the U.S. Food and Drug Administration (FDA) for any indication. Genetic eye diseases are particularly suitable candidates for RNA therapies given the potential to cure these conditions using gene editing tools like CRISPR/Cas9 (*23*). Such cargoes are challenging to fit within the size constraints of adeno-associated viral (AAV) vectors, the most widely used vehicle for ocular gene delivery (*24*), but can be packaged as RNAs into nanoparticles to achieve in vivo editing (*25*, *26*). Nanoparticle delivery of gene editing enzymes additionally avoids their long-term expression, limiting the off-target effects and immune reactions that can occur when these molecules are expressed from AAV vectors (*27*–*29*).

**Fig. 1.**
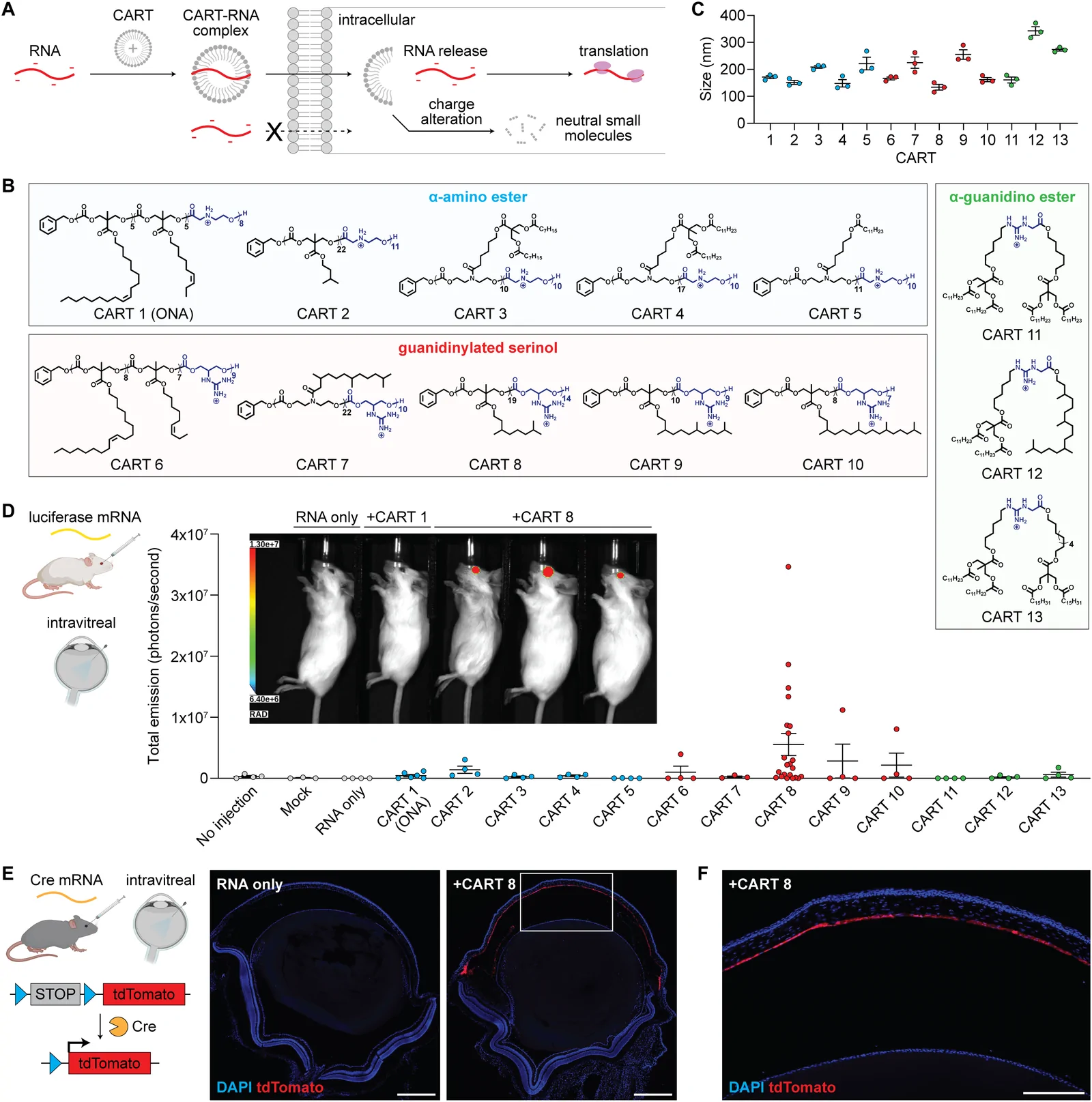
Intravitreal screening identifies CARTs enabling ocular delivery of mRNA. **A**) Proposed mechanism of CART delivery. Cationic CARTs electrostatically complex polyanionic RNA, protecting it during cell delivery. Once inside cells, CARTs undergo self-degradation into neutral small molecules, releasing the RNA into the cytosol. (**B**) Chemical structures of CARTs. Structures are grouped by their charge-altering motif (α-amino ester, guanidinylated serinol, or α-guanidino ester). (**C**) Particle size of CARTs complexed with 420 ng of luciferase mRNA at a 10:1 charge ratio in acidified 1x PBS (pH 5.5). Data are mean ± SEM. *n* = 3 technical replicates per group. (**D**) In vivo luminescence imaging of BALB/c mice without treatment or 12–16 hours after mock injection, intravitreal injection of luciferase mRNA only, or intravitreal injection of luciferase mRNA with the indicated CART. Eyes were dosed with 300 ng of RNA at a 10:1 charge ratio. Data are mean ± SEM. *n* = 3–22 eyes per group. (**E**) Whole eye sections from Ai9 mice 1 week after intravitreal injection of Cre mRNA only or Cre mRNA with CART 8. Eyes were dosed with 300 ng of RNA at a 10:1 charge ratio. *n* = 4–6 eyes per group. Scale bars, 500 μm. (**F**) Anterior segment section from an Ai9 mouse 1 week after intravitreal injection of Cre mRNA with CART 8. Image location is indicated by white inset in (E). Image is representative of *n* = 2 of 6 eyes. Scale bar, 200 μm. Created in BioRender. Wang, S. (2026) https://BioRender.com/pg8axe3.

Previously, we described a class of biodegradable polymers called charge-altering releasable transporters (CARTs) that efficiently deliver RNA in vivo (*6*, *30*–*33*). At acidic pH (<5.5), cationic CARTs electrostatically bind polyanionic RNA, forming stable nanoparticle complexes (Fig. 1A). Upon administration, these nanoparticles rapidly enter cells and self-degrade into neutral small molecules via a charge-altering mechanism, releasing the RNA into the cytosol. The tropism of CARTs can be tuned by modifying their chemical structures, allowing for targeted transfection of organs such as the spleen (*30*, *31*), lungs (*32*, *34*), and muscle (*35*, *36*). More recently, CARTs have also been prepared as discrete entities termed DIGITs, which enable RNA delivery to red blood cells (*37*). Importantly, CARTs exhibited minimal toxicity in mice after intravenous or intramuscular delivery as measured by serum cytokine levels and histopathology (*31*, *35*), suggesting that they may be safe to use in patients.

Here, we report the discovery of a CART that selectively transfects the corneal endothelium, a non-regenerative cell layer essential for maintaining corneal clarity. Genetic mutations in this cell type are responsible for several blinding diseases including Fuchs’ endothelial dystrophy (*38*), the leading cause of corneal transplantation worldwide (*39*). We characterize the safety profile of CART-RNA nanoparticles in mouse eyes and show that they facilitate the delivery of diverse RNA cargoes to the corneal endothelium. We further demonstrate CART transfection of corneal endothelial cells from a human donor in vitro and in a non-human primate (NHP) in vivo, providing a proof of concept for translating this system to patients. Our findings establish CARTs as a platform for non-viral gene delivery to the eye, with the potential to treat corneal dystrophies and other vision disorders.

## RESULTS

### Screening of CARTs for ocular delivery of mRNA

To determine if CARTs could transfect the eye, we synthesized a panel of 13 CARTs (Fig. 1B and fig. S1, A and B) spanning three distinct charge-altering motifs, each associated with a different mechanism of charge-cancellation and RNA release (fig. S2A) (*6*, *32*, *37*). This panel included ONA (CART 1), our most studied CART (*30*), as well as CARTs containing α-amino esters (CARTs 2–5) (*31*, *36*), guanidinylated serinols (CARTs 6–10) (*32*), or α-guanidino esters (CARTs 11–13) (*37*). It also encompassed a diverse selection of lipid moieties (unsaturated lipids for CART 1 and 6; isoprenoid lipids for CART 2, 7–10, and 12; fork lipids for CART 3, 4, and 11–13) and two types of polymeric backbones (e.g. CART 7 versus CART 9) to investigate the effects of hydrophobic substituents and lipid spacing (*30*, *31*, *36*). CARTs were formulated with mRNA encoding firefly luciferase by micropipette mixing, yielding average particle diameters of 134–343 nm (Fig. 1C). Polydispersity indices ranged from 0.1–0.3, indicating mostly uniform size distributions (fig. S2B), and all complexes carried a net positive surface charge (fig. S2C). For in vivo testing, we injected CART-RNA formulations into the vitreous cavity of the eye, a common route for ocular drug delivery.

We performed intravitreal injections of CARTs complexed with luciferase mRNA in albino mice and imaged their eyes after 12–16 hours. Formulations using α-amino or α-guanidino ester CARTs showed little to no change in luminescence compared to untreated, mock injected, or RNA only controls (Fig. 1D). In contrast, in a subset of eyes injected with guanidinylated serinol CARTs, we observed strong luciferase activity, suggesting that this group of CARTs might facilitate ocular delivery of mRNA. We focused on CART 8 (BnO-MTCCit19-GSer14), which exhibited the highest luminescence, lowest surface charge, and most rapid RNA release kinetics among guanidinylated serinol CARTs (fig. S2, D to H), and used Ai9 mice to assess its tropism in the eye. Following exposure to Cre recombinase, these animals constitutively express the tdTomato fluorescent protein (*40*), allowing identification of transfected cells. TdTomato was not detected in eyes that received Cre mRNA only but was robustly expressed in two of six eyes after intravitreal injection of Cre mRNA with CART 8 (Fig. 1E). Surprisingly, this expression was absent from the retina and instead localized to structures in the anterior segment, including the posterior surface of the cornea (Fig. 1F).

### Transfection of the corneal endothelium by CART 8

We hypothesized that introducing CART 8 closer to the cornea would enhance the efficiency and consistency of corneal transfection. To test this, we first trialed topical delivery in Ai9 mice by placing a 5 μL drop of CART 8 complexed with Cre mRNA directly onto the ocular surface. However, this route of administration failed to produce any tdTomato-positive cells in the cornea (fig. S3). We therefore explored intracameral delivery by injecting CARTs into the anterior chamber of the eye, granting them immediate access to the cornea and other anterior segment structures. Such injections are routinely performed in patients and can be done in a clinic setting using only topical anesthesia. As a control, we also formulated Cre mRNA into LNPs containing SM-102, the ionizable lipid used in the Moderna COVID-19 vaccine (*41*).

**Fig. 2.**
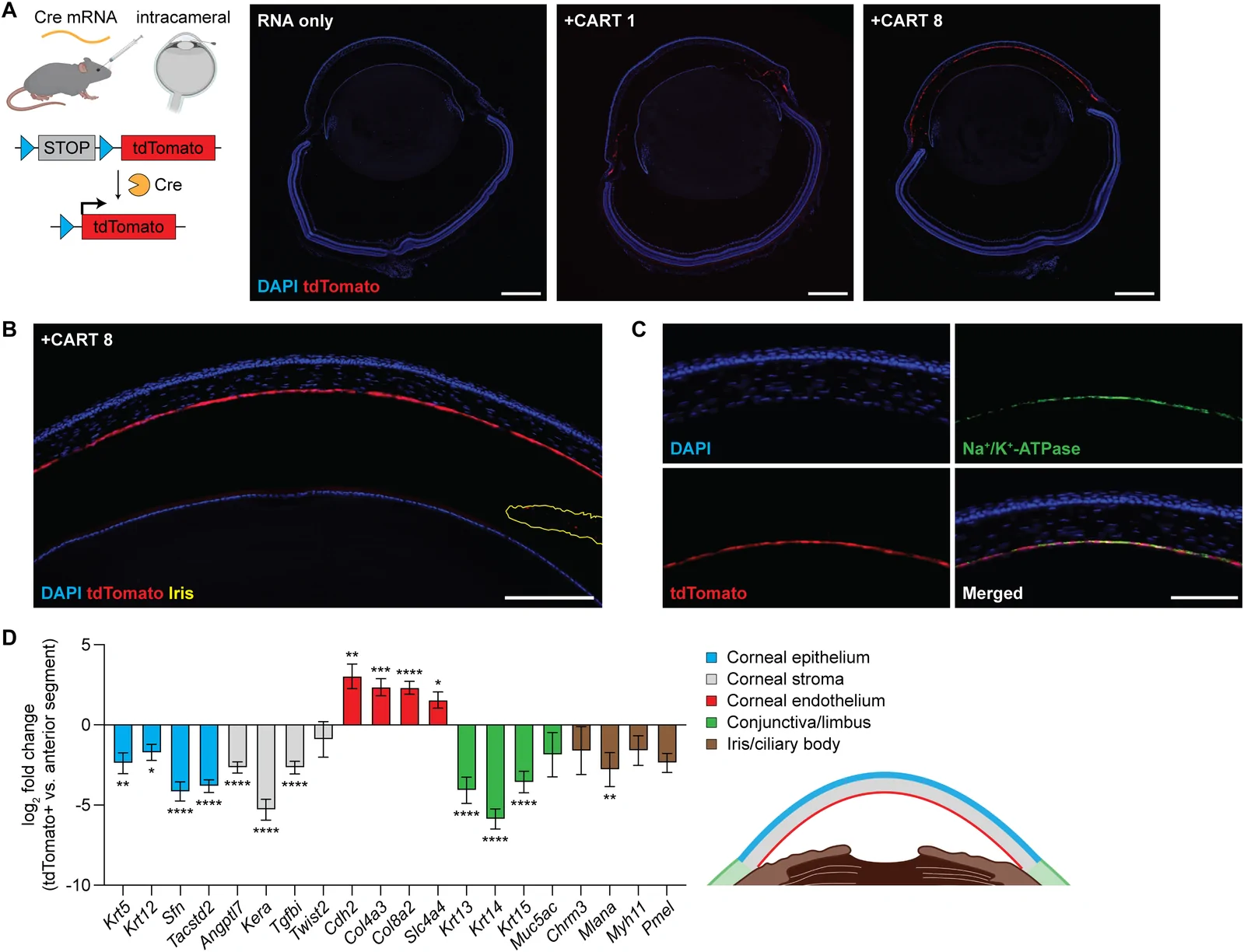
Intracameral CART 8 selectively transfects the corneal endothelium. **A**) Whole eye sections from Ai9 mice 1 week after intracameral injection of Cre mRNA only, Cre mRNA with CART 1, or Cre mRNA with CART 8. Eyes were dosed with 300 ng of RNA at a 10:1 charge ratio. *n* = 4 eyes per group. Scale bars, 500 μm. (**B**) Anterior segment section from an Ai9 mouse 1 week after intracameral injection of Cre mRNA with CART 8. Iris structures are outlined in yellow. *n* = 4 eyes. Scale bar, 200 μm. (**C**) Immunostaining for Na^+^/K^+^-ATPase in a corneal section from an Ai9 mouse 1 week after intracameral injection of Cre mRNA with CART 8. *n* = 2 eyes. Scale bar, 100 μm. (**D**) Up- and down-regulation of cell type-specific genes in tdTomato-positive anterior segment cells from Ai9 mice 1 week after intracameral injection of Cre mRNA with CART 8. TdTomato-positive cells were compared to cells from untreated anterior segments by RNA-seq. Colors denote different anterior segment cell types. Data are mean ± SEM. *n* = 3–6 eyes per group. * adjusted *P* < 0.05, ** adjusted *P* < 0.01, *** adjusted *P* < 0.001, **** adjusted *P* < 0.0001. Created in BioRender. Wang, S. (2026) https://BioRender.com/pg8axe3.

Intracameral delivery of Cre mRNA with CART 8 induced tdTomato expression in the corneas of Ai9 mice (Fig. 2A). In contrast, tdTomato was absent from eyes that received Cre mRNA only and was restricted to the iris and iridocorneal angle after injection of Cre mRNA with CART 1 or SM-102 LNP (fig. S4A). Corneal expression of tdTomato was additionally observed in eyes that received CART 6 or CART 9, two of the other guanidinylated serinol CARTs (fig. S4B). While CART 1, CART 6, and CART 10 showed partial transfection of the iris (figs. S4B and S5A), iris transfection with CART 8 was much more sparse (fig. S5B). Given tdTomato labeling of the posterior corneal surface (Fig. 2B), we reasoned that CART 8 transfected the corneal endothelium. Consistent with this, tdTomato expression in the cornea colocalized with immunostaining for Na^+^/K^+^-ATPase (Fig. 2C), a marker of corneal endothelial cells (*42*).

To evaluate the cell type-specificity of CART 8, we sorted tdTomato-positive cells from the anterior segments of Ai9 mice after intracameral injection of Cre mRNA with CART 8 (fig. S6A) and profiled their gene expression by RNA sequencing (RNA-seq). Compared to untreated anterior segments, tdTomato-positive cells were significantly enriched for corneal endothelial marker genes such as *Cdh2*, *Col4a3*, and *Slc4a4* (*43*, *44*). Conversely, tdTomato-positive cells were significantly depleted for markers of the corneal epithelium (*45*), corneal stroma (*43*, *45*, *46*), conjunctiva and limbus (*43*, *45*), and iris and ciliary body (*47*) (Fig. 2D and data S1). Together, these data demonstrate selective transfection of the corneal endothelium by CART 8 following intracameral administration.

### Safety of intracameral CART 8 in mice

We previously reported that guanidinylated serinol CARTs facilitate RNA delivery to the spleen and lungs (*32*). To determine if systemic transfection occurred following intracameral injection of CART 8, we harvested spleens and lungs from Ai9 mice. No tdTomato-positive cells were detected in these organs after administration of Cre mRNA with or without CART 8 (fig. S6, B and C), suggesting that the tropism of intracameral CART 8 was confined to the eye.

Our prior work also showed that adjusting the cation-to-anion charge ratio of CART-mRNA formulations can modify their transfection efficiency (*6*, *31*, *32*). We thus tested intracameral injections of CART 8 with Cre mRNA at different cation (guanidinium)-to-anion (phosphate) ratios using a fixed dose of Cre mRNA. Corneal flat mounts from Ai9 mice exhibited robust transfection of the endothelium at as low as a 1.25:1 charge ratio (fig. S7, A and B), with reduced transfection observed at much lower or higher ratios. TdTomato expression at this charge ratio was sparse in the iris and absent from the ciliary body (fig. S7C), indicating that CART 8 was still highly selective for the corneal endothelium.

**Fig. 3.**
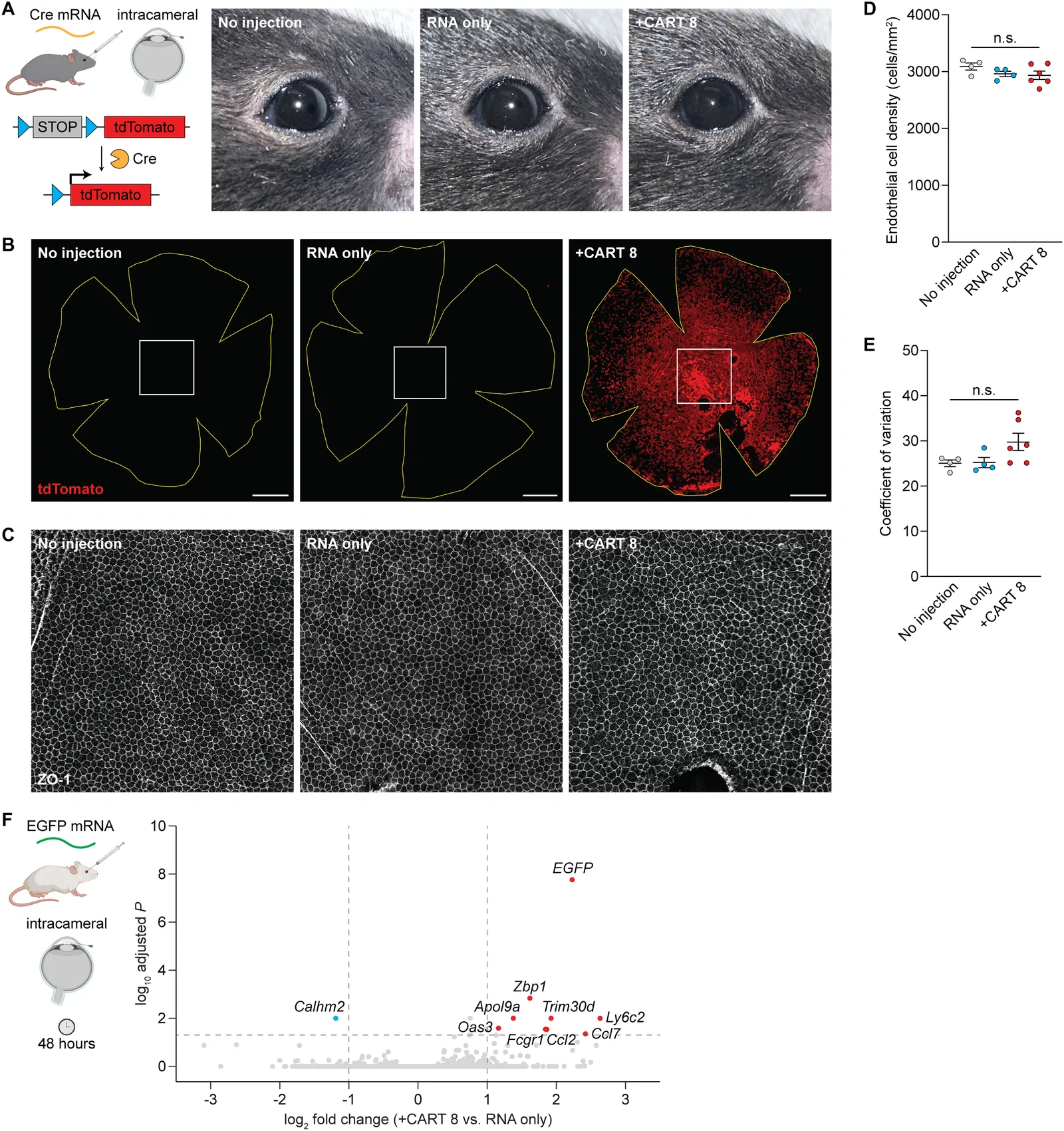
Effect of intracameral CART 8 on corneal morphology and gene expression. **A**) External photos of Ai9 mice without treatment or 1 week after intracameral injection of Cre mRNA only or Cre mRNA with CART 8. Eyes were dosed with 500 ng of RNA at a 1.25:1 charge ratio. *n* = 4–6 eyes per group. (**B**) Representative images of corneal flat mounts from Ai9 mice without treatment or 1 week after intracameral injection of Cre mRNA only or Cre mRNA with CART 8. *n* = 4–6 eyes per group. Scale bars, 500 μm. (**C**) Immunostaining for ZO-1 in corneal flat mounts from Ai9 mice without treatment or 1 week after intracameral injection of Cre mRNA only or Cre mRNA with CART 8. Image locations are indicated by white insets in (B). *n* = 4–6 eyes per group. (**D** and **E**) Quantification of corneal endothelial cell density (D) and coefficient of variation (E) in anti-ZO-1-stained corneal flat mounts. Data are mean ± SEM. *n* = 4–6 eyes per group. n.s., not significant. (**F**) Differentially expressed genes in anterior segments from C57BL/6 J mice 48 hours after intracameral injection of EGFP mRNA with CART 8 compared to EGFP mRNA only. Eyes were dosed with 500 ng of RNA at a 1.25:1 charge ratio. Red and blue dots denote significantly (absolute log2 fold change ≥1, adjusted *P* < 0.05) up- and down-regulated genes by RNA-seq, respectively. *n* = 4–6 eyes per group. Created in BioRender. Wang, S. (2026) https://BioRender.com/pg8axe3.

Using a 1.25:1 charge ratio, we characterized the safety of intracameral CART 8 in mice by assessing its effect on corneal morphology and gene expression. At a gross level, eyes that received Cre mRNA with CART 8 displayed no obvious changes in appearance such as corneal edema, buphthalmos, or cataract formation (Fig. 3A). To measure the health of corneal endothelial cells, we performed immunostaining for zonula occludens-1 (ZO-1) to delineate cell junctions (*48*) (Fig. 3, B and C) and quantified endothelial cell density and size variation in the central cornea (fig. S8). We found no significant difference in endothelial cell density at one week or four months following injection of Cre mRNA with CART 8 relative to Cre mRNA alone (Fig. 3D and fig. S9, A and B). While there was a trend toward more variable cell size in eyes that received Cre mRNA with CART 8, this effect did not reach statistical significance at either time point (Fig. 3E and fig. S9B). In contrast, examination of corneas one week after injection of CART 8 without any RNA cargo revealed increased variability in endothelial cell size (fig. S9, D and E), possibly due to excess positive charge in the absence of RNA (fig. S9F). CART 8 may therefore trigger corneal endothelial toxicity when not complexed with polyanions.

We then conducted RNA-seq of mouse anterior segments 48 hours after intracameral injection of mRNA encoding enhanced green fluorescent protein (EGFP) with or without CART 8. As expected, EGFP mRNA was strongly up-regulated in eyes that received CART 8 (Fig. 3F and data S2), corroborating our sequencing results. Compared to injection of EGFP mRNA alone, addition of CART 8 significantly (absolute log2 fold change ≥1, adjusted *P* < 0.05) altered the expression of only nine endogenous genes, most of which possess immune functions (*49*–*53*). Importantly, intracameral CART 8 did not affect the expression of corneal endothelial genes (*Cdh2*, *Col4a3*, *Col8a2*, *Slc4a4*) (*43*, *44*), canonical pro-inflammatory cytokines (*Il1a*, *Il1b*, *Il6*, *Tnf*) (*54*), or markers of cell death (*Bax*, *Bcl2*, *Casp3*, *Fas*) (*55*). Supporting this, measurement of intraocular cytokines following delivery of EGFP mRNA with CART 8 compared to EGFP mRNA alone detected up-regulation of several chemokines, but no increases in IL-1α, IL-1β, IL-6 or TNF-α (fig. S10A). Likewise, we found no significant difference in corneal terminal deoxynucleotidyl transferase dUTP nick end labeling (TUNEL) staining 48 hours after injection of luciferase mRNA with or without CART 8 (fig. S9, G and H).

To evaluate the persistence of gene expression changes following CART 8 administration, we assayed three of the up-regulated genes in anterior segments four months after intracameral delivery of Cre mRNA with or without CART 8. Quantitative polymerase chain reaction (qPCR) at this time point showed similar levels of *Ly6c2*, *Trim30d*, and *Zbp1* in both conditions (fig. S9C), suggesting that induction of these genes was transient.

### CART 8 delivery of circular RNA and CRISPR/Cas9

**Fig. 4.**
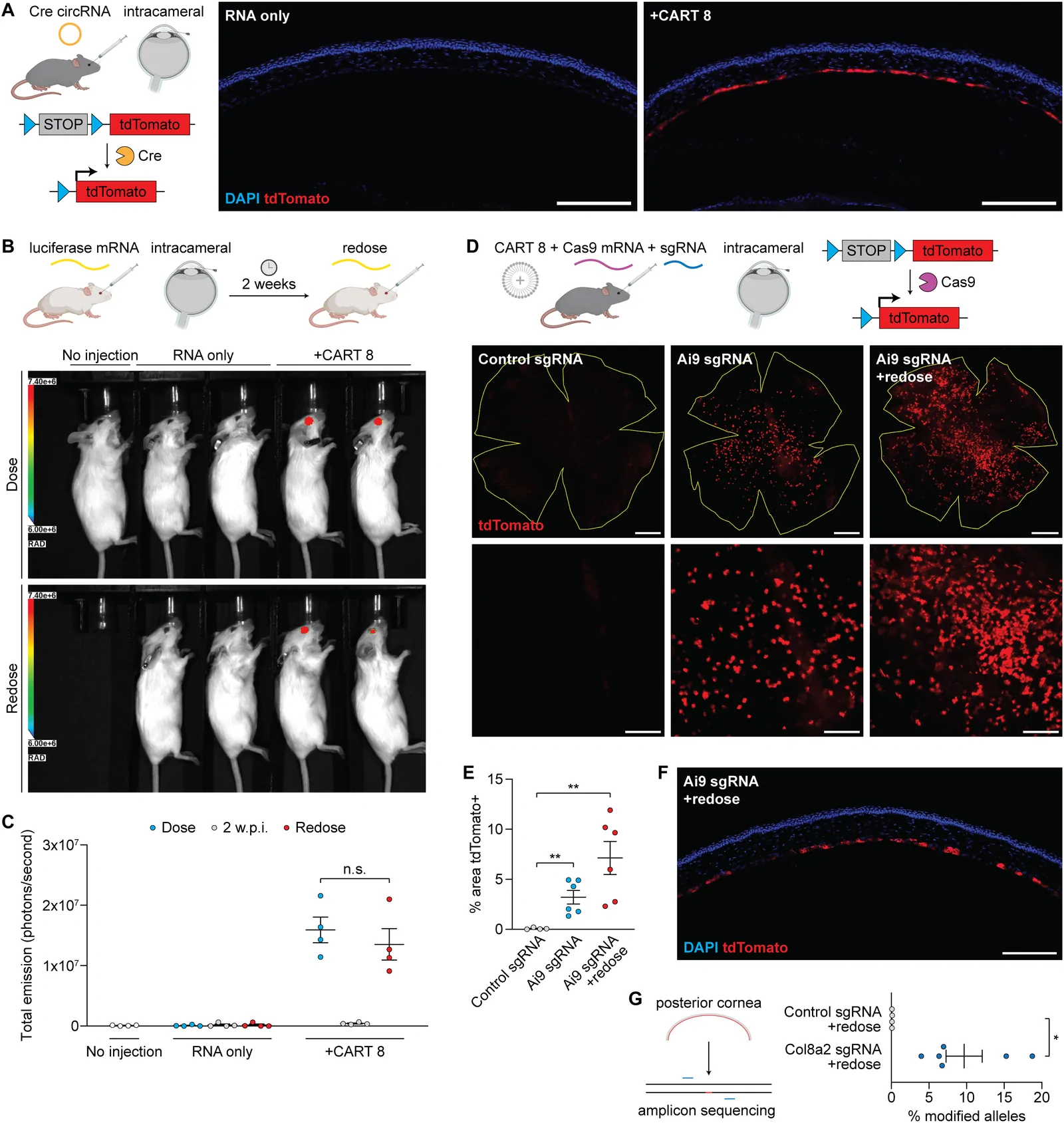
CART 8 enables circular RNA expression and gene editing in the corneal endothelium. **A**) Anterior segment sections from Ai9 mice 1 week after intracameral injection of Cre circRNA only or Cre circRNA with CART 8. Eyes were dosed with 500 ng of RNA at a 1.25:1 charge ratio. *n* = 4 eyes per group. Scale bars, 200 μm. (**B**) In vivo luminescence imaging of BALB/c mice without treatment or 16 hours after intracameral dosing or redosing of luciferase mRNA only or luciferase mRNA with CART 8. Eyes were dosed with 500 ng of RNA at a 1.25:1 charge ratio. Redosing was performed after 2 weeks. *n* = 4 eyes per group. (**C**) Quantification of in vivo luminescence imaging. Data are mean ± SEM. *n* = 4 eyes per group. n.s., not significant. (**D**) Representative images of corneal flat mounts from Ai9 mice 1 week after intracameral dosing or redosing of Cas9 mRNA and the indicated sgRNA with CART 8. Eyes were dosed with 600 ng of total RNA at a 5:1 charge ratio. Redosing was performed after 1 week. *n* = 4–6 eyes per group. Scale bars, 500 μm (top), 100 μm (bottom). (**E**) Quantification of CRISPR/Cas9-mediated gene editing in Ai9 corneal flat mounts. Data are mean ± SEM. *n* = 4–6 eyes per group. **P < 0.01 by two-tailed Student’s *t* test. (**F**) Corneal section from an Ai9 mouse 1 week after intracameral redosing of Cas9 mRNA and Ai9 sgRNA with CART 8. *n* = 2 eyes. Scale bar, 200 μm. (**G**) Quantification of CRISPR/Cas9-mediated gene editing in C57BL/6 J mice at the *Col8a2* locus 1 week after intracameral redosing of Cas9 mRNA and the indicated sgRNA with CART 8. Data are mean ± SEM. *n* = 4–6 eyes per group. *P < 0.05 by two-tailed Student’s *t* test. Created in BioRender. Wang, S. (2026) https://BioRender.com/pg8axe3.

Circular RNAs (circRNAs) represent an emerging class of RNA therapeutics exhibiting enhanced stability and durable expression (*33*). To determine if CART 8 could deliver circRNA to the corneal endothelium, we synthesized a circRNA encoding Cre recombinase. Cre circRNA resisted digestion by the exoribonuclease RNase R (fig. S11), validating its circular structure. Following intracameral injection in Ai9 mice, we detected tdTomato expression in the corneal endothelium of eyes that received Cre circRNA with CART 8, but not Cre circRNA only (Fig. 4A). These data indicate that CART 8 can deliver both mRNA and circRNA to corneal endothelial cells.

One advantage of RNA delivery systems is the potential for repeated administration. To confirm the feasibility of redosing CART 8, we performed serial intracameral injections of luciferase mRNA in albino mice. Consistent with our intravitreal experiments, eyes that received luciferase mRNA with CART 8 showed robust luciferase activity after 16 hours, while eyes that received luciferase mRNA only showed no change in luminescence (Fig. 4, B and C). Following resolution of luciferase activity by two weeks post-injection, we repeated intracameral delivery of luciferase mRNA with or without CART 8. At 16 hours after redosing, we observed luciferase activity comparable to initial levels, demonstrating that intracameral CART 8 could be administered more than once. Concentrations of intraocular cytokines following redosing were also highly similar to those measured after the first injection (fig. S10B), supporting the safety of repeated delivery.

We further tested if CART 8 was able to mediate corneal gene editing by complexing CART 8 with mRNA encoding the Cas9 nuclease and a single guide RNA (sgRNA) targeting the STOP cassette in Ai9 or Ai14 mice (Ai9 sgRNA) (*56*). Successful CRISPR/Cas9 editing deletes this STOP cassette, enabling tdTomato expression. As a control, we injected CART 8 and Cas9 mRNA with a sgRNA targeting Gal4, a gene not found in mice (*56*). We employed equal masses of Cas9 mRNA and sgRNA in all formulations as was previously reported for CRISPR/Cas9 LNPs (*25*, *57*).

Initial attempts at CART 8 delivery of CRISPR/Cas9 using a 1.25:1 charge ratio led to only modest editing (fig. S12, A and B), potentially due to larger and more varied particle sizes (fig. S13) that prompted us to trial a higher charge ratio. Following intracameral injection of CART 8 complexed with Cas9 mRNA and Ai9 sgRNA at a 5:1 charge ratio, 1.3–4.9% of the cornea by area was tdTomato-positive, indicating successful gene editing (Fig. 4, D and E). In contrast, negligible tdTomato expression was seen after injection of Cas9 mRNA and the control sgRNA with CART 8. To increase editing, we administered a second dose of Cas9 mRNA and Ai9 sgRNA with CART 8 one week after the initial injection, resulting in tdTomato expression in 2.3–11.9% of the cornea. Sections through these eyes revealed confinement of tdTomato labeling to the posterior corneal surface (Fig. 4F), verifying that editing took place in corneal endothelial cells. Analysis of tdTomato expression at one month after dosing additionally confirmed the persistence of edited cells (fig. S12, C and D).

We wondered if CART 8 could be applied to edit a disease-associated gene in the cornea. Previously, Uehara *et al.* reported that CRISPR/Cas9-mediated disruption of *Col8a2* in corneal endothelial cells rescues mice with early-onset Fuchs’ endothelial dystrophy (*58*). As a proof of concept, we performed two intracameral injections of CART 8 complexed with Cas9 mRNA and a sgRNA targeting the *Col8a2* start codon in wild-type mice and measured editing at this locus using amplicon sequencing. *Col8a2* editing was detected in 4.0–18.8% of alleles in posterior corneas that received the Col8a2 sgRNA, compared to only 0.1% of alleles in those that received the control sgRNA (Fig. 4G and fig. S12E). Collectively, these findings highlight the ability of CART 8 to deliver diverse RNA cargoes to the corneal endothelium, including CRISPR/Cas9 for gene editing.

### CART 8 activity in translational models

**Fig. 5.**
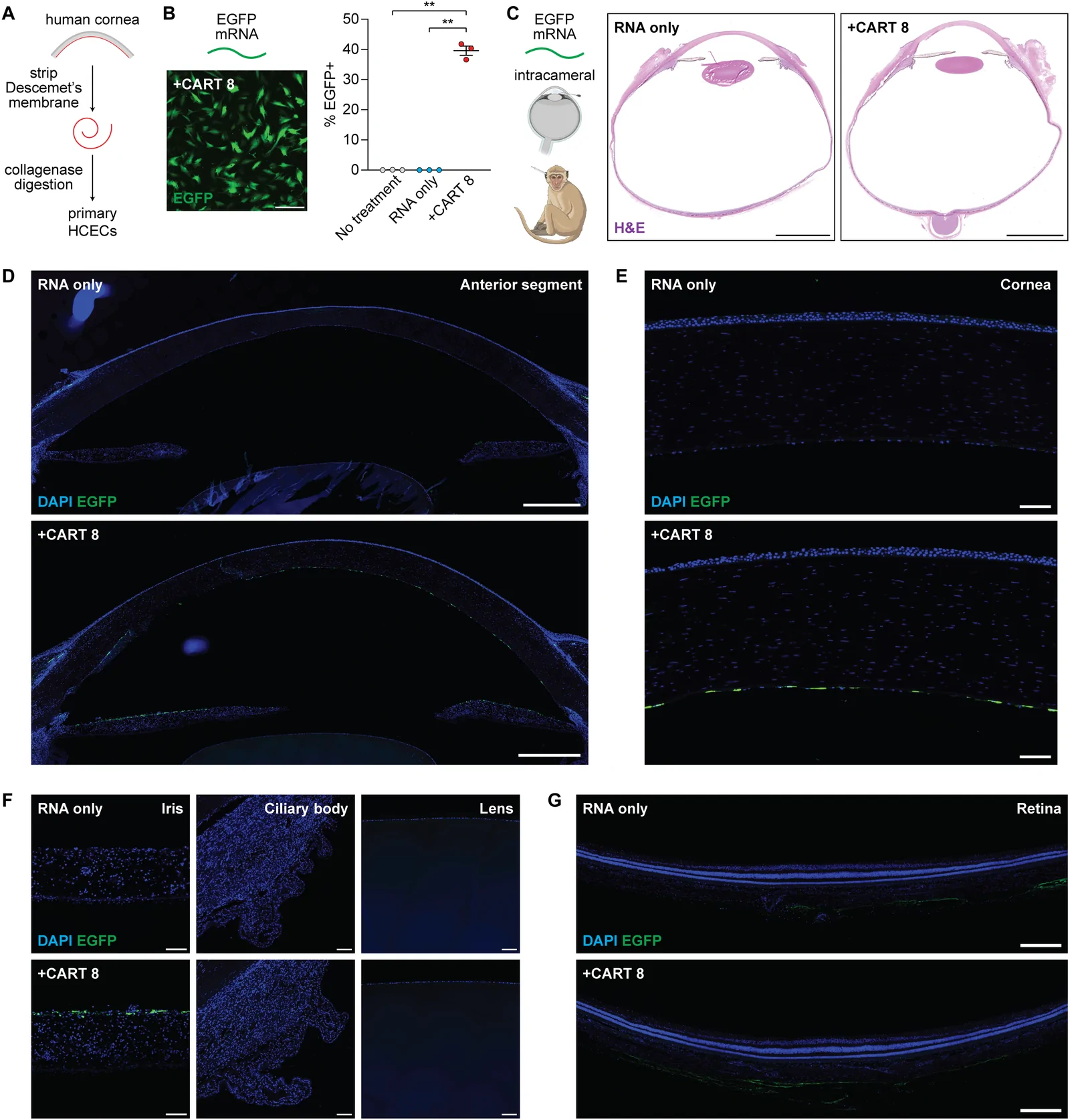
CART 8 transfects corneal endothelial cells from a human donor and in a non-human primate. **A**) Isolation of primary human corneal endothelial cells (HCECs). Descemet’s membrane and the attached corneal endothelium were stripped from a donor cornea and digested with collagenase to liberate HCECs. Isolated cells were cultured for a maximum of three passages. (**B**) Left: Representative image of HCECs 16 hours after treatment with EGFP mRNA with CART 8. Scale bar, 200 μm. Right: Flow cytometry quantification of EGFP expression in HCECs without treatment or 16 hours after treatment with EGFP mRNA only or EGFP mRNA with CART 8. Cells were treated with 100 ng of RNA per 96-well at a 4:1 charge ratio. Data are mean ± SEM. *n* = 3 biological replicates per group. **P < 0.01 by two-tailed Student’s *t* test. (**C**) Hematoxylin and eosin (H&E) staining of whole eye sections from an NHP 48 hours after intracameral injection of EGFP mRNA only or EGFP mRNA with CART 8. Eyes were dosed with 20 μg of RNA at a 3:2 charge ratio. *n* = 1 eye per group. Scale bars, 5 mm. (**D** to **G**) Immunostaining for EGFP in the indicated tissues of NHP eyes 48 hours after intracameral injection of EGFP mRNA only or EGFP mRNA with CART 8. *n* = 1 eye per group. Scale bars, 1 mm (D), 100 μm (E and F), 500 μm (G). Created in BioRender. Wang, S. (2026) https://BioRender.com/pg8axe3.

We lastly explored if the functionality of CART 8 might translate to humans by evaluating its activity in primary human corneal endothelial cells (HCECs). These cells were isolated from a donor cornea and minimally passaged to best approximate their in vivo physiology (Fig. 5A). Following administration of EGFP mRNA with CART 8, we detected EGFP expression in HCECs (Fig. 5B and fig. S14, A to D), demonstrating transfection of these cells. Conversely, no expression was seen in untreated HCECs or those that received EGFP mRNA alone. HCECs showed no signs of reduced viability at 16 hours after treatment with CART 8-RNA complexes (fig. S14E). These results suggest that CART 8 may enable RNA delivery to the corneal endothelium not only in mice, but also in humans.

Using HCECs, we investigated the effect of small molecule inhibitors of clathrin-mediated endocytosis (chlorpromazine) (*59*), caveolae-mediated endocytosis (methyl-β-cyclodextrin) (*60*), macropinocytosis (EIPA) (*61*), cell-surface glycosaminoglycans (sodium chlorate) (*62*), and endosomal acidification (bafilomycin A) (*63*) on CART 8 transfection in vitro. Of these inhibitors, methyl-β-cyclodextrin produced the largest reduction in transfection efficiency (fig. S15A), implicating caveolae-mediated endocytosis as a major contributor to RNA delivery by CART 8. To determine if CART 8 administration might elicit neutralizing antibodies, we also measured HCEC transfection in the presence of plasma from treated or untreated mice. Plasma from mice that received intracameral injections of EGFP mRNA with CART 8 did not significantly affect transfection relative to plasma from control animals (fig. S15B), arguing against the formation of neutralizing antibodies.

We then assessed the tropism of CART 8 in an NHP model, the African green monkey. One eye was intracamerally injected with CART 8 complexed with 20 μg of EGFP mRNA in a 50 μL volume, while the other was injected with EGFP mRNA only. After 48 hours, eyes were collected for histology including hematoxylin and eosin (H&E) staining (Fig. 5C) and immunostaining for EGFP. Initial examination revealed EGFP signal near the limbus and posterior wall of both eyes even in the absence of primary antibody (fig. S16, A and B), consistent with autofluorescence from extraocular tissues. In the eye that received CART 8, we observed EGFP expression in corneal endothelial cells on the posterior corneal surface (Fig. 5, D and E). EGFP-positive cells were additionally found in the anterior iris, but not in the ciliary body, lens, or retina (Fig. 5, F and G). Although the overall structure of the eye was maintained following injection of CART 8, slight numbers of mixed polymorphonuclear and mononuclear cells were identified at the iridocorneal angle (fig. S16C), indicating mild anterior chamber inflammation. In summary, our findings confirm the ability of CART 8 to transfect the corneal endothelium in an NHP in vivo, supporting the clinical development of this delivery technology.

## DISCUSSION

RNA therapies hold tremendous promise for treating genetic diseases. However, their application to vision disorders is still in its infancy, largely due to the lack of non-viral delivery platforms that can target specific ocular cell types. In this study, we identified a CART that delivers RNA selectively to the corneal endothelium, enabling therapeutic interventions such as gene augmentation and gene editing in these non-regenerative cells. CART 8 moreover transfected primary HCECs and the corneal endothelium in an NHP, underscoring its potential to deliver RNA to the same cell layer in patients. Our findings are especially relevant for sight-threatening diseases of the corneal endothelium, including Fuchs’ endothelial dystrophy, posterior polymorphous corneal dystrophy, congenital hereditary endothelial dystrophy, and iridocorneal endothelial syndrome (*64*), opening new treatment avenues for these conditions outside of corneal transplantation. Endothelial cell transfection could additionally be useful during cataract surgery and other intraocular procedures to express genes that protect against endothelial cell loss (*65*) or to secrete factors that expedite postoperative healing (*66*).

Studies of LNPs in the eye have demonstrated RNA delivery to the retina, retinal pigment epithelium, and trabecular meshwork following intravitreal, subretinal, or intracameral administration (*67*–*70*). However, transfection of the corneal endothelium by LNPs remains inefficient (*70*), even when nanoparticles are injected directly into the corneal stroma (*71*). Although adenoviral vectors can successfully deliver genes to corneal endothelial cells (*58*, *72*), their translation to patients faces multiple challenges, including pre-existing immunity against common serotypes and the potential to induce substantial inflammation (*73*, *74*). While not as severe, ocular inflammation from LNPs has also been described, as evidenced by activation of retinal microglia and T cell infiltration in the choroid after subretinal delivery in an NHP (*68*). Here, we observed up-regulation of several chemokines and immune-related genes following CART 8 administration in mice and mild anterior chamber inflammation in an NHP, but no changes in corneal endothelial genes, canonical pro-inflammatory cytokines, or markers of cell death. In clinical applications, we anticipate that concurrent use of topical corticosteroids may further mitigate the safety risks of CART 8.

When delivered without a polyanionic counterpart, CART 8 led to greater variability in endothelial cell size, suggestive of toxicity to the corneal endothelium. This finding is not surprising as highly cationic molecules can destabilize cell membranes, triggering cell necrosis and inflammation (*75*, *76*). Moreover, positively charged polymers and lipids such as polyethylenimine (PEI) and 1,2-dioleoyl-3-trimethylammonium propane (DOTAP) have been shown to impair Na^+^/K^+^-ATPase (*76*), which is abundantly expressed on corneal endothelial cells. In contrast, intracameral injection of CART 8-RNA complexes did not cause any significant morphological abnormalities. While there was a trend toward increased variation in endothelial cell size one week after injection of Cre mRNA with CART 8 compared to Cre mRNA only, this difference was less apparent at four months, supporting the long-term safety of CART 8 for RNA delivery.

Several limitations of our study should be acknowledged. Although we evaluated the effect of CART 8 on corneal endothelial cell density and morphology, we did not directly measure endothelial cell function. Parameters for gene editing including the sgRNA-to-Cas9 ratio and charge ratio were also not fully optimized, and we did not verify protein level changes after *Col8a2* editing or screen for off-target CRISPR/Cas9 activity. Previously, Uehara *et al.* reported that an editing rate of 23.7% was sufficient to restore corneal endothelial function in the *Col8a2* mouse model of Fuchs’ endothelial dystrophy (*58*). Follow-up experiments will be needed to determine if CART 8-mediated gene editing could similarly rescue corneal disease. We additionally demonstrated RNA delivery to the corneal endothelium in an NHP, but these results were limited to a single animal and not quantitatively assessed. Furthermore, unlike in mice, CART 8 transfected the anterior iris of the NHP. Given that iris tissue is highly vascular and rich in immune cells (*77*), this off-target expression could potentially introduce immune-related safety concerns. Finally, we did not examine the cumulative impact of repeated CART 8 administration on the corneal endothelium. Such data would be particularly relevant for diseased or injured corneas, where an already compromised endothelium might be less able to tolerate successive treatments. The efficiency of CART 8 transfection in these conditions may also differ from that in healthy eyes. It will thus be essential to test the therapeutic capabilities of CART 8 in an animal model of corneal endothelial dystrophy or damage prior to use in patients.

Why does CART 8 transfect the corneal endothelium? Although the exact reasons for endothelial cell selectivity remain to be determined, we hypothesize that the tropism of CART 8 is largely conferred by its guanidinylated serinols. In our initial screen, nearly every eye that exhibited luciferase activity had received a guanidinylated serinol CART, suggesting this charge-altering motif to be necessary for endothelial cell entry. Consistent with these observations, intracameral injection of CART 6 and CART 9, two other guanidinylated serinol CARTs, also resulted in endothelial cell transfection. However, guanidinylated serinols alone are not sufficient for endothelial cell targeting, as intracameral CART 10 did not transfect the corneal endothelium. One possibility is that the lower surface charge and faster RNA release kinetics of CART 8 relative to other guanidinylated serinol CARTs may have facilitated more efficient delivery of RNA. This delivery was strongly inhibited by methyl-β-cyclodextrin in vitro, supporting a dependence on caveolae-mediated endocytosis. The low frequency of luciferase expression with guanidinylated serinol CARTs in our initial screen likely reflected limited exposure to the cornea following intravitreal administration. Furthermore, negatively charged polymers in the vitreous such as hyaluronic acid might have trapped CART-RNA complexes (*78*, *79*), preventing them from reaching posterior segment structures like the retina. Conversely, CART transfection after intracameral dosing may have benefited from natural convection currents in the anterior chamber, which circulate warmer fluid near the lens and iris toward the corneal endothelium (*80*). Future optimization of CARTs using these principles may enable RNA delivery to additional ocular cell types for gene therapy and gene editing applications.

## MATERIALS AND METHODS

### CART synthesis

CARTs were synthesized as previously described (*6*, *30*–*32*, *36*, *37*). α-amino ester and guanidinylated serinol CARTs were synthesized as di- or tri-block polymers via anionic ring opening polymerization in Boc protected form and purified by dialysis against methanol. α-guanidino ester CARTs were synthesized in Boc protected form and purified via flash column chromatography (*37*). Boc protected CARTs were deprotected with 20% trifluoroacetic acid in dichloromethane for 4 hours, dried, and dissolved in dimethyl sulfoxide (15–40 mM cationic concentration) before use.

### CART-RNA formulations

CARTs were complexed with RNA in acidified 1x PBS (pH 5.5) containing 20% sucrose by rapid mixing using a micropipette for 20 seconds immediately prior to administration. Modified mRNAs encoding luciferase (L-7202), Cre (L-7211), EGFP (L-8101), and Cas9 (L-7206) were obtained from TriLink BioTechnologies. For size and zeta potential measurements, CART-RNA formulations were diluted with 1 mL of deionized water, immediately transferred to a disposable clear plastic cuvette, and measured using a NanoBrook Omni analyzer (Brookhaven Instruments). RNA doses and cation (ammonium or guanidinium)-to-anion (phosphate) charge ratios for all experiments are provided in figure legends.

### RNA encapsulation and release assays

RNA encapsulation and release were measured using Quant-iT RiboGreen RNA Reagent (Thermo Fisher Scientific) in black 96-well plates. To quantify encapsulation, CART-RNA formulations were added to TE buffer (pH 5.5) containing 1:100 of RiboGreen fluorescent dye and fluorescence measured using a plate reader (480 nm excitation, 530 nm emission, 140 ms integration time). To quantify RNA release, CART-RNA formulations mixed with 10 μL of 100 mM Na_2_CO_3_ were added to TE buffer (pH 7.5) containing 1:100 of RiboGreen fluorescent dye and fluorescence measured as above. Standard curves in TE buffers were used to calculate the percentage of RNA that was non-encapsulated or released.

### Mouse injections

Mouse experiments were performed in 1–6-month-old BALB/c (#000651), Ai9 (#007909), C57BL/6 J (#000664), and Ai14 (#007914) mice obtained from The Jackson Laboratory. Both male and female mice were used and were randomly assigned to treatment groups. For intravitreal injections, animals were anesthetized using 2–3% inhaled isoflurane and pupils dilated with topical 1% tropicamide. A sharp 30-gauge needle was then inserted 1 mm posterior to the limbus to create a sclerotomy, through which a blunt 33-gauge Hamilton needle was introduced and 2 μL of volume administered under direct visualization. Care was taken to ensure that the needle did not damage the lens or retina. For intracameral injections, mice were anesthetized as above. A microinjector pressure system (Picospritzer III) with a sharpened, pulled glass micropipette in a micromanipulator was used to inject reagents intracamerally through the cornea 1 mm anterior to the limbus, and 1 μL of volume was administered under direct visualization without disturbing intraocular structures. Topical antibiotic ointment was applied to the eyes to aid in recovery. All mouse experiments were approved by the Institutional Animal Care and Use Committee at Stanford University (APLAC-14046).

### In vivo imaging

In vivo luciferase activity was measured using an Ami HT optical imaging system (Spectral Instruments). Mice were anesthetized as above and injected intraperitoneally with 100 μL of 30 mg/mL D-luciferin (Invitrogen). Mice were imaged after 5 minutes using default settings and an exposure time of 1 second. Luminescent activity was quantified using Aura 4.0 imaging software with equal-sized regions of interest centered on each eye.

External photos of mouse eyes were captured using an iPhone 14 Pro (Apple) with 3x magnification and flash on.

### LNP formulation

SM-102 LNP was formulated by rapidly mixing SM-102 (Avanti), DSPC (Avanti), cholesterol (Avanti), and DMG-PEG 2000 (Avanti) at a 50:10:38.5:1.5 molar ratio with mRNA using a micropipette. RNA doses and charge ratios for experiments are provided in figure legends.

### Mouse histology

Mouse eyes for cryosections were enucleated 1 week after injection. Following intravitreal injections, eyes were directly submerged in 4% paraformaldehyde (PFA) for 4 hours at room temperature. Fixed globes were subsequently washed with PBS, cryoprotected in a sucrose gradient, and embedded in a 1:1 mixture of 30% sucrose in PBS and optimal cutting temperature compound (Tissue-Tek) on dry ice. Following intracameral injections, eyes were first dissected in PBS to remove excess orbital tissue and a sclerotomy created before fixation in 4% PFA for 2 hours at room temperature. Fixed globes were then washed, cryoprotected, and embedded as above. Frozen globes were cut on a cryostat into 20-μm sections, stained with 0.1 μg/mL of 4′,6-diamidino-2-phenylindole (DAPI) (Invitrogen) to label nuclei, and mounted onto glass slides using ProLong Gold Antifade (Invitrogen). To detect Na^+^/K^+^-ATPase, sections were additionally blocked with PBS containing 5% normal donkey serum (Jackson ImmunoResearch) and 0.1% Triton X-100 (Sigma-Aldrich) for 1 hour at room temperature, stained with 1:100 of rabbit anti-Na+/K + -ATPase antibody (Invitrogen, MA5–32184) in blocking buffer overnight at 4°C, and incubated with 1:1000 of Alexa Fluor 488 donkey anti-rabbit secondary antibody (Jackson ImmunoResearch, 711–545-152) in PBS for 2 hours at room temperature.

Mouse eyes for corneal flat mounts were enucleated 1 week after injection unless otherwise specified. Using curved Vannas scissors, eyes were cut circumferentially at the limbus to isolate the cornea, which was fixed in 2% PFA for 30 minutes at room temperature. After PBS washes, corneas were relaxed with 4 radial incisions, carefully flattened onto glass slides with the endothelium facing up, and mounted using ProLong Gold Antifade. To detect ZO-I, corneas were additionally blocked with PBS containing 5% normal donkey serum and 0.1% Triton X-100 for 1 hour at room temperature, stained with 1:100 of rabbit anti-ZO-I antibody (Invitrogen, 61–7300) in blocking buffer overnight at 4°C, and incubated with 1:1000 of Alexa Fluor 488 donkey anti-rabbit secondary antibody in PBS for 2 hours at room temperature.

### Microscopy

Images of mouse cryosections and corneal flat mounts were acquired on either a Keyence BZ-X fluorescence microscope (2x, 10x, or 20x air objective) or Zeiss LSM 880 confocal microscope (10x air objective). All image analysis was performed using ImageJ. Anti-ZO-1-stained corneal endothelial cells were segmented and quantified using a custom ImageJ plugin in which the central 750 x 750 μm region of each flat mount was subjected to automated background subtraction, thresholding, despeckling, watershedding, and particle counting. Endothelial cell density was calculated by dividing 10^6^ by the mean cell area in μm^2^. Coefficient of variation was calculated by dividing the standard deviation of cell areas by their mean. Gene editing in Ai9 and Ai14 corneal flat mounts was quantified using a custom ImageJ plugin in which each flat mount was subjected to automated background subtraction followed by thresholding to define tdTomato-positive cells. Editing was calculated as the percent area of each cornea that was tdTomato-positive. TUNEL staining in corneal flat mounts was quantified using a custom ImageJ plugin in which the central 750 x 750 μm region of each flat mount was subjected to automated background subtraction, thresholding, despeckling, and particle counting. Custom ImageJ plugins are available at https://zenodo.org/records/19344868.

### Flow cytometry and cell sorting

Fluorescence-activated cell sorting (FACS) of tdTomato-positive cells was performed on a BD FACSAria II. Eyes from Ai9 mice were cut circumferentially 1 mm posterior to the limbus to isolate the cornea, iris, and part of the ciliary body. To obtain single-cell suspensions, isolated tissues were digested with 625 units/mL of collagenase type I (Sigma-Alrich) in PBS for 45 minutes at 37°C followed by 0.25% Trypsin-EDTA (Gibco) for 10 minutes at 37°C (*81*), then washed with PBS and stained with 0.1 μg/mL of DAPI to exclude dead cells. TdTomato-positive cells were collected from eyes that received Cre mRNA with CART 8. Two eyes were pooled for each sample and a minimum of 500 cells sorted. Anterior segment control cells were collected from eyes without treatment. One eye was used for each sample and 1,000 cells sorted.

Flow cytometry of mouse spleen, mouse lung, and HCECs was performed on an Attune NxT flow cytometer. Isolated spleens were mashed into a cell suspension, filtered through a 70 μm cell strainer, treated with RBC Lysis Buffer (Tonbo) to lyse red blood cells, washed with PBS, and stained with 0.1 μg/mL of DAPI. Isolated lungs were first digested with 8 units/mL of DNase I (New England Biolabs) and 125 units/mL of collagenase type I in PBS for 45 minutes at 37°C, then processed identically to spleens. HCECs were dissociated using 0.05% trypsin-EDTA (Gibco), washed with PBS, and stained with 0.1 μg/mL of DAPI. All FACS and flow cytometry data were analyzed using FlowJo 10.

### RNA isolation and sequencing

For RNA-seq of sorted cells, total RNA was collected using a Quick-RNA Microprep Kit (Zymo) and sent to Azenta Life Sciences (South Plainfield, NJ, USA) for ultra-low input RNA-seq library construction and sequencing with a target depth of 20 million reads per sample. The resulting FASTQ files were mapped to the mm10 genome using Bowtie2 (version 2.3.4.1), filtered for a MAPQ score > 10 using SAMtools (version 1.16.1), and subjected to PCR duplicate removal using Picard MarkDuplicates to generate BAM files (*82*–*84*). Gene expression counts were then obtained from BAM files using HTSeq (version 2.0.1) and differentially expressed genes identified using DESeq2 (version 1.26.0) (*85*, *86*).

For RNA-seq and qPCR of anterior segments, eyes were cut circumferentially 1 mm posterior to the limbus to isolate the cornea, iris, and part of the ciliary body. Tissues were homogenized using a handheld pellet pestle (Kimble Chase) in 300 μL of RNA Lysis Buffer (Zymo) and centrifuged at 16,000 x g for 5 minutes. Total RNA was then collected from the supernatant using a Quick-RNA Microprep Kit (Zymo) following manufacturer’s instructions. For RNA-seq, RNA was sent to Azenta Life Sciences for library construction and sequencing, mapped to a custom mm10 genome containing the EGFP sequence, and analyzed as described above. For qPCR, cDNA was synthesized using HiScript IV All-in-One Ultra RT SuperMix (Vazyme) and amplified using SupRealQ Ultra Hunter SYBR qPCR Master Mix (Vazyme) on a CFX96 real-time PCR detection system (Bio-Rad). Reactions were performed in triplicate with expression normalized to the housekeeping gene *Gapdh*. Sequences for qPCR primers are listed in table S1.

### TUNEL assay

TUNEL staining was performed using the Click-iT Plus TUNEL Assay for In Situ Apoptosis Detection (Invitrogen) following manufacturer’s instructions. For DNase I treatment, corneas from untreated mice were fixed, permeabilized, and incubated with 1 unit of DNase I for 30 minutes at room temperature.

### Cytokine measurements

Anterior segments were isolated as described above and placed in 100 μL per sample of RIPA lysis and extraction buffer (Thermo Fisher Scientific) containing protease and phosphatase inhibitors (Thermo Fisher Scientific). Following sonication and centrifugation to remove debris, the supernatant was collected and submitted to the Human Immune Monitoring Center at Stanford University for cytokine profiling. Cytokine levels were measured using mouse 48-plex Procarta kits (Thermo Fisher Scientific) following manufacturer’s instructions. Each sample was measured in duplicate, and plates were read using a Luminex 200 or a FM3D FlexMap instrument with a lower bound of 50 beads per sample per cytokine.

### Circular RNA synthesis

Cre circRNA was synthesized using methods previously described (*33*). Briefly, the Cre coding sequence was cloned into the pRC0940 plasmid backbone and PCR amplified to synthesis the circRNA template. In vitro transcription was performed using a HiScribe T7 High Yield RNA Synthesis Kit (New England Biolabs) with 5% substitution of 5-methylcytidine triphosphate (TriLink BioTechnologies) for cytidine triphosphate. Column purified RNA was digested with 0.5 units of RNase R (Biosearch Technologies) per microgram of RNA for 30 minutes at 37°C to isolate circRNA.

### Guide RNAs

Ai9 (5′-AAGTAAAACCTCTACAAATG-3′), control (5′-AACGACTAGTTAGGCGTGTA-3′), and Col8a2 (5′-CCCATCCACAGACGCCATGC-3′) sgRNAs with standard 2′-O-methyl and 3′ phosphorothioate modifications were obtained from Synthego and resuspended at a concentration of 1.6 μg/μL in acidified 1x PBS (pH 5.5) containing 20% sucrose. A 1:1 mass ratio of Cas9 mRNA to sgRNA was used in all formulations.

### Amplicon sequencing

The posterior cornea (corneal endothelium, Descemet’s membrane, and posterior stroma) was mechanically separated from mouse corneas and processed using a Quick-DNA Microprep Kit (Zymo) following manufacturer’s instructions to isolate genomic DNA. Nested PCR was performed using outer primers for 8 cycles followed by inner primers for 30 cycles to amplify the *Col8a2* locus (table S1). Amplicons were sent to Azenta Life Sciences for Amplicon-EZ sequencing with a target depth of 50,000 reads per sample. The resulting FASTQ files were analyzed using CRISPResso2 with default settings (*87*). Modified alleles were defined as those with any addition, deletion, or substitution within a 40-bp window centered 3 bp upstream of the protospacer adjacent motif (PAM) sequence.

### Human corneal endothelial cell culture

Primary HCECs were isolated as previously described (*88*) from a 44-year-old male donor cornea procured by the San Diego Eye Bank under protocols approved by the Eye Bank Association of America. Briefly, Descemet’s membrane and the attached corneal endothelium were stripped from the stroma and incubated in 0.2% collagenase type II (Worthington Biochemical) in PBS for 1 hour at 37°C. Cells were then pelleted and resuspended in endothelial cell growth medium 2 (EGM-2) with premixed supplements (PromoCell). Cells were grown on plates coated with FNC Coating Mix (Athena Enzyme Systems) at 37°C and 5% CO_2_ for a maximum of three passages and sub-cultured using 0.05% trypsin-EDTA (Gibco).

In vitro transfection of HCECs was performed in a 96-well flat bottom plate at 40–60% confluency. For each well, culture medium was reduced to 25 μL of EGM-2 and 2 μL of EGFP mRNA with or without CART 8 introduced. After two hours, 100 μL of EGM-2 was added. CART 8 was complexed with EGFP mRNA in acidified 1x PBS (pH 5.5) containing 20% sucrose. Small molecule inhibitors (chlorpromazine, methyl-β-cyclodextrin, EIPA, sodium chlorate, and bafilomycin A) were obtained from Sigma-Aldrich and added to cell culture media 30 minutes before transfection, except for sodium chlorate which was added 48 hours prior. Cells were imaged on an EVOS M5000 microscope (Invitrogen) using a 10x air objective.

### Neutralizing antibody assay

Blood was collected from the retro-orbital sac of mice using a 31-gauge insulin syringe and mixed with PBS containing 5 mM of EDTA to prevent clotting. The resulting mixture was centrifuged at 2,000 x g for 10 minutes and the plasma supernatant collected. Plasma from two mice was combined per condition and added to cell culture media 30 minutes before transfection.

### NHP injections

Intracameral injections were performed in an adult male African green monkey (*Chlorocebus sabaeus*). The animal was anesthetized with intramuscular ketamine (8 mg/kg) and xylazine (1.6 mg/kg) and pupils dilated with topical 10% phenylephrine and 1% cyclopentolate. Topical 0.5% proparacaine was then administered, an eye speculum placed, and the ocular surface rinsed with 5% Betadine solution followed by sterile 0.9% saline. Intracameral injection was performed in each eye using a 31-gauge insulin syringe (UltiCare VetRx U-100) inserted through the temporal cornea 2 mm anterior to the limbus. A volume of 50 μL containing 20 μg of EGFP mRNA with or without CART 8 was administered under direct visualization without disturbing intraocular structures. Topical antibiotic ointment was applied to the eyes to aid in recovery. All NHP experiments were approved by the Institutional Animal Care and Use Committee of the St. Kitts Biomedical Research Foundation (AC24256–004).

### NHP histology

The animal was euthanized and eyes enucleated 48 hours after injection. Following removal of excess orbital tissue, eyes were submerged in Davidson’s fixative for 24 hours at room temperature, washed with PBS, and trimmed horizontally. Tissues were subsequently dehydrated through gradient alcohols, cleared in xylene, and infiltrated with paraffin wax for embedding. Paraffin-embedded tissue blocks were cut on a microtome into 4-μm sections and either stained with hematoxylin and eosin, or 1:1000 of rabbit monoclonal anti-GFP antibody (Abcam, ab183734) for 1 hour at ambient temperature. Binding of the primary antibody was visualized with Discovery OmniMap anti-rabbit HRP (Roche, 760–4311) and a Cy5 fluorophore (Roche, 760–238) on a Ventana Discovery Ultra immunostainer (Roche). For some slides, primary anti-GFP antibody was omitted as a negative control. Whole slides were imaged on a Pannoramic SCAN II digital slide scanner (3DHisTech) using 20x objective magnification.

### Statistical analysis

Group data are shown as mean ± SEM. Two-tailed Student’s *t* tests were used to compare experimental groups and were performed using GraphPad Prism. *P* < 0.05 was considered statistically significant.
