# Supplementary material for: RNA delivery to the corneal endothelium using charge-altering releasable transporters
Source: Sci Adv. 2026 Aug 21;12(34):eady8161. doi: 10.1126/sciadv.ady8161 (PMC13496210; doi:10.1126/sciadv.ady8161)
Supplement: Supplementary file 1 [file sciadv.ady8161_sm.pdf]

Science Advances
AAAS

Supplementary Materials for

RNA delivery to the corneal endothelium using charge-altering releasable transporters

Sean K. Wang *et al.*

Corresponding author: Paul A. Wender, wenderp@stanford.edu; Howard Y. Chang, howchang@stanford.edu

Sci. Adv. **12**, eady8161 (2026)
DOI: 10.1126/sciadv.ady8161

The PDF file includes:

Figs. S1 to S16
Table S1
Legends for data S1 and S2

Other Supplementary Material for this manuscript includes the following:

Data S1 and S2

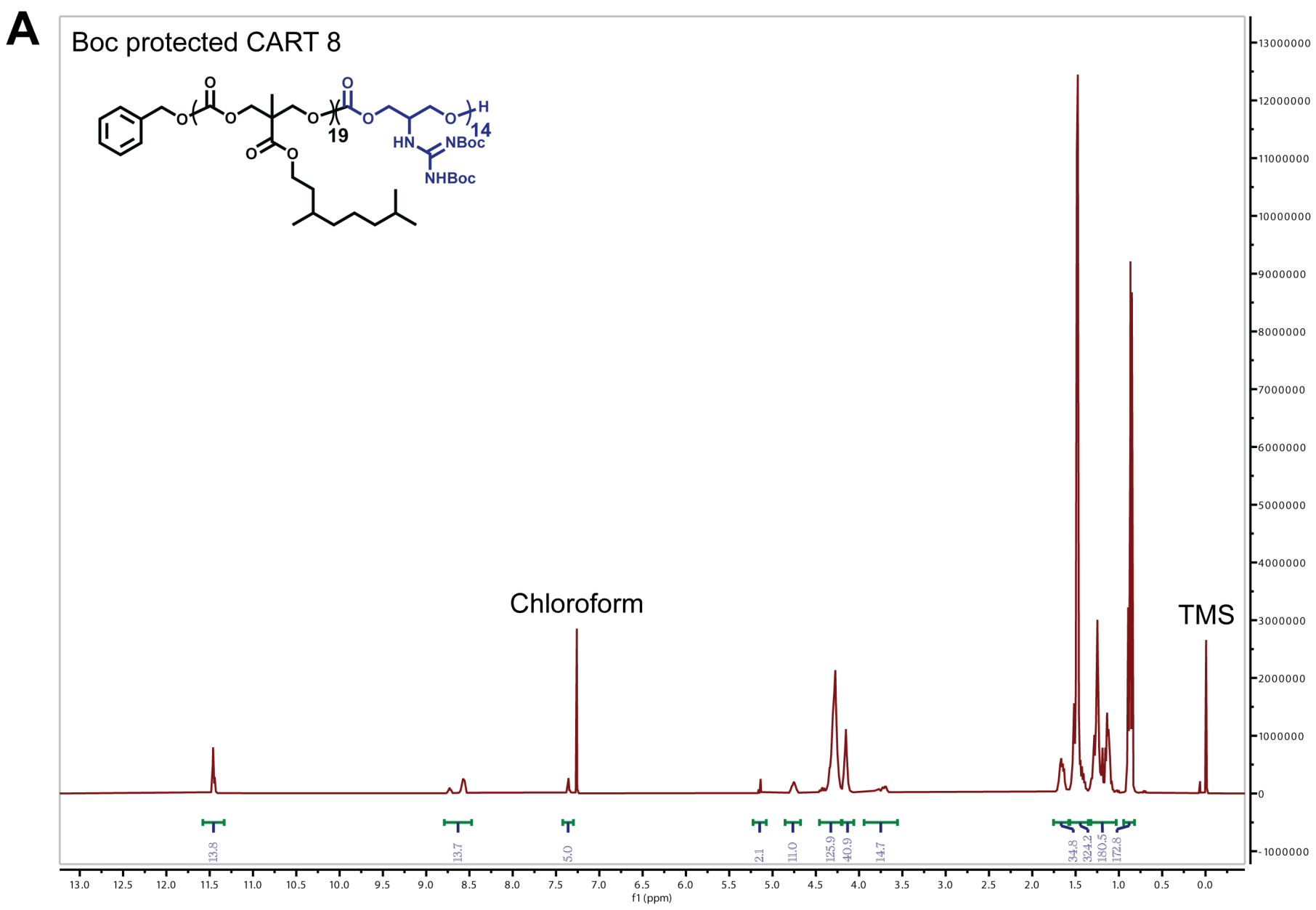

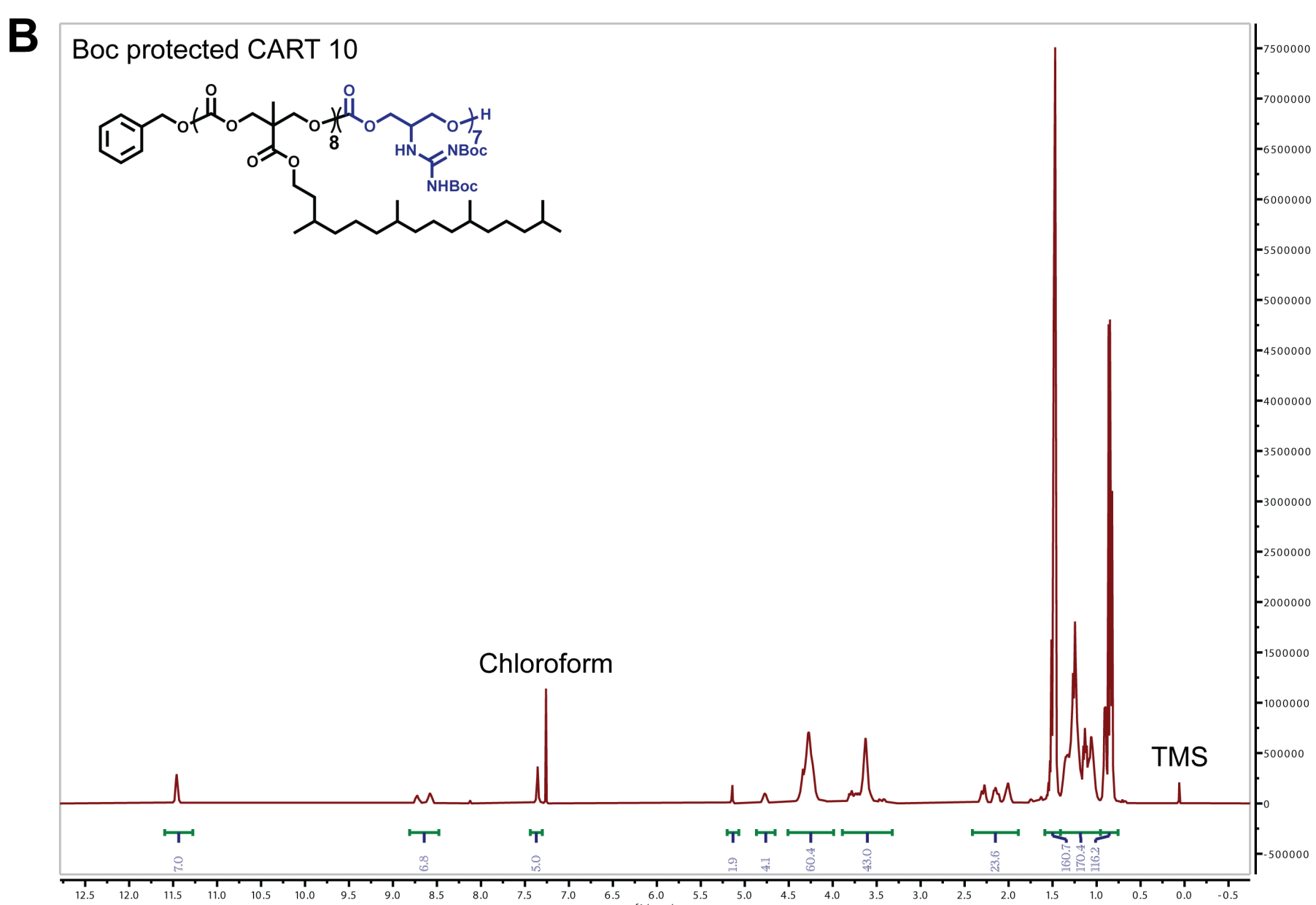

Figure S1. Proton nuclear magnetic resonance spectra.

(A,B) ^{1}H nuclear magnetic resonance (NMR) spectra for Boc-protected CART 8 (A) and CART 10 (B). Tetramethylsilane (TMS) was used as the internal standard. ^{1}H NMR spectra for CARTs 1, 2, 3, 4, 5, 6, 7, 9, 11, 12, and 13 have been previously reported (*6*, *31*, *32*, *36*, *37*).

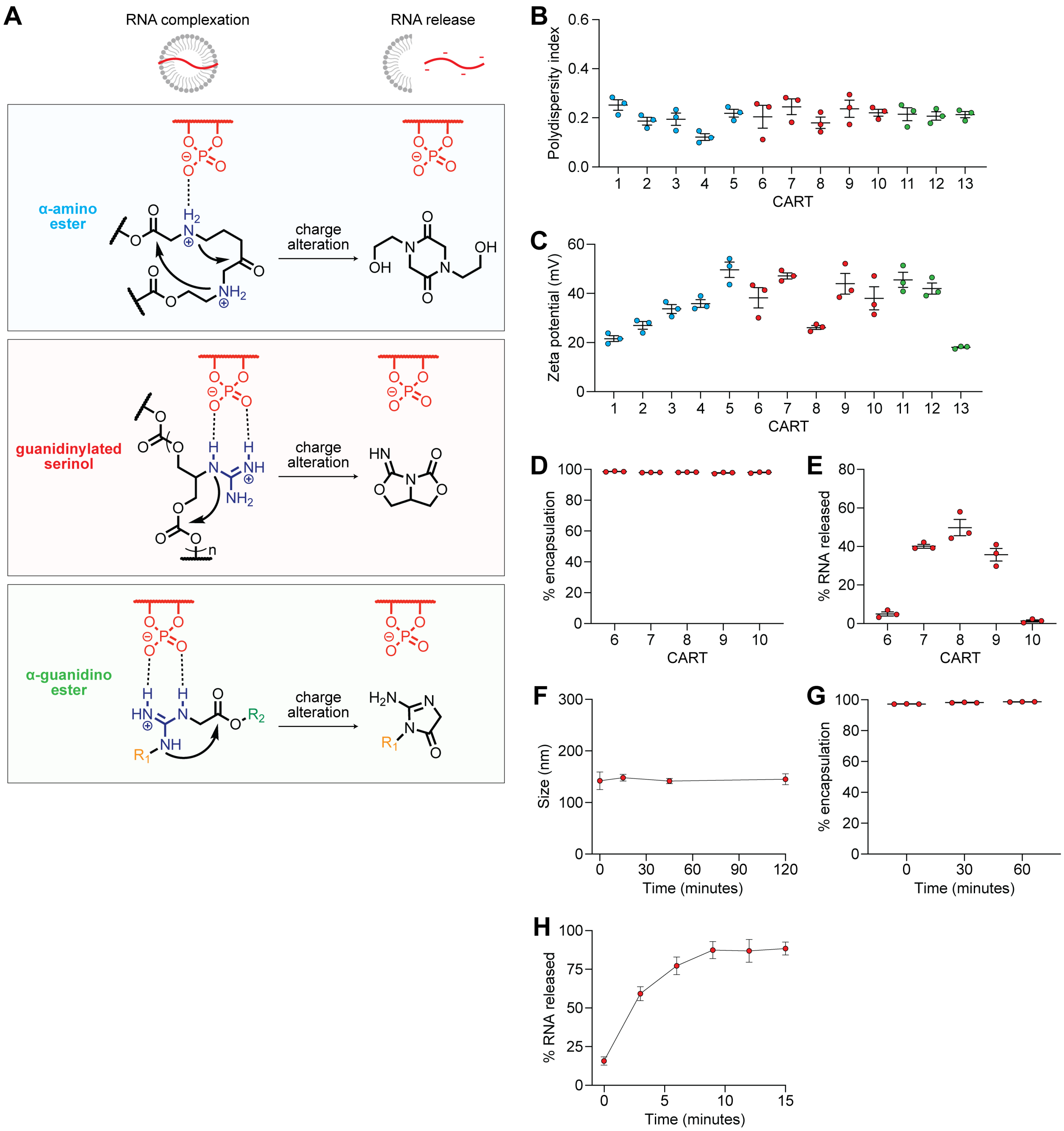

Figure S2. CART charge alteration mechanisms and quality control metrics.

(A) Proposed RNA release mechanisms of CARTs with indicated charge-altering motifs.

(B-E) Polydispersity index (B), surface charge (C), encapsulation rate (D), and RNA release profiles (E) of the indicated CARTs complexed with 0.42-1 μg of luciferase mRNA at a 10:1 cation (ammonium or guanidinium)-to-anion (phosphate) charge ratio in acidified 1x PBS (pH 5.5). RNA release was measured 2 minutes after addition of Na_2CO_3. Data are mean ± SEM. n = 3 technical replicates per group.

(F-H) Size (F), encapsulation rate (G), and RNA release profiles (H) at the indicated time points after formulation of CART 8 with 1 μg of luciferase mRNA at a 10:1 charge ratio in acidified 1x PBS. For RNA release, Na_2CO_3 was added at time = 0. Data are mean ± 95% CI for (F), mean ± SEM for (G) and (H). n = 3 technical replicates per group.

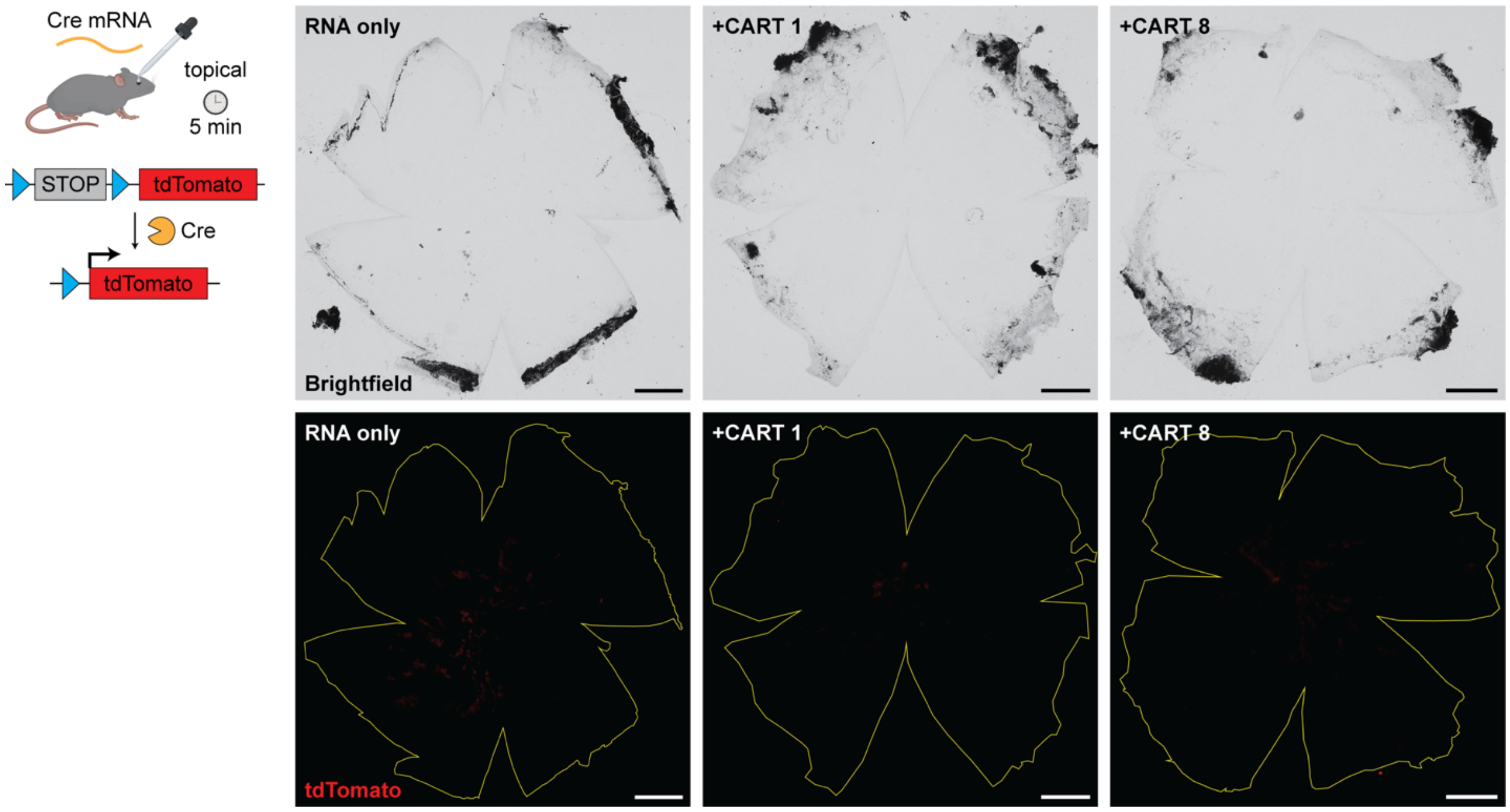

Figure S3. Topical administration of CARTs.

Representative images of corneal flat mounts from Ai9 mice 1 week after topical administration of Cre mRNA only, Cre mRNA with CART 1, or Cre mRNA with CART 8. Eyes were dosed with 1 ug of RNA at a 10:1 charge ratio. A drop volume of 5 µl was placed on each eye and allowed to remain for 5 minutes. n = 4 eyes per group. Scale bars, 500 µm.

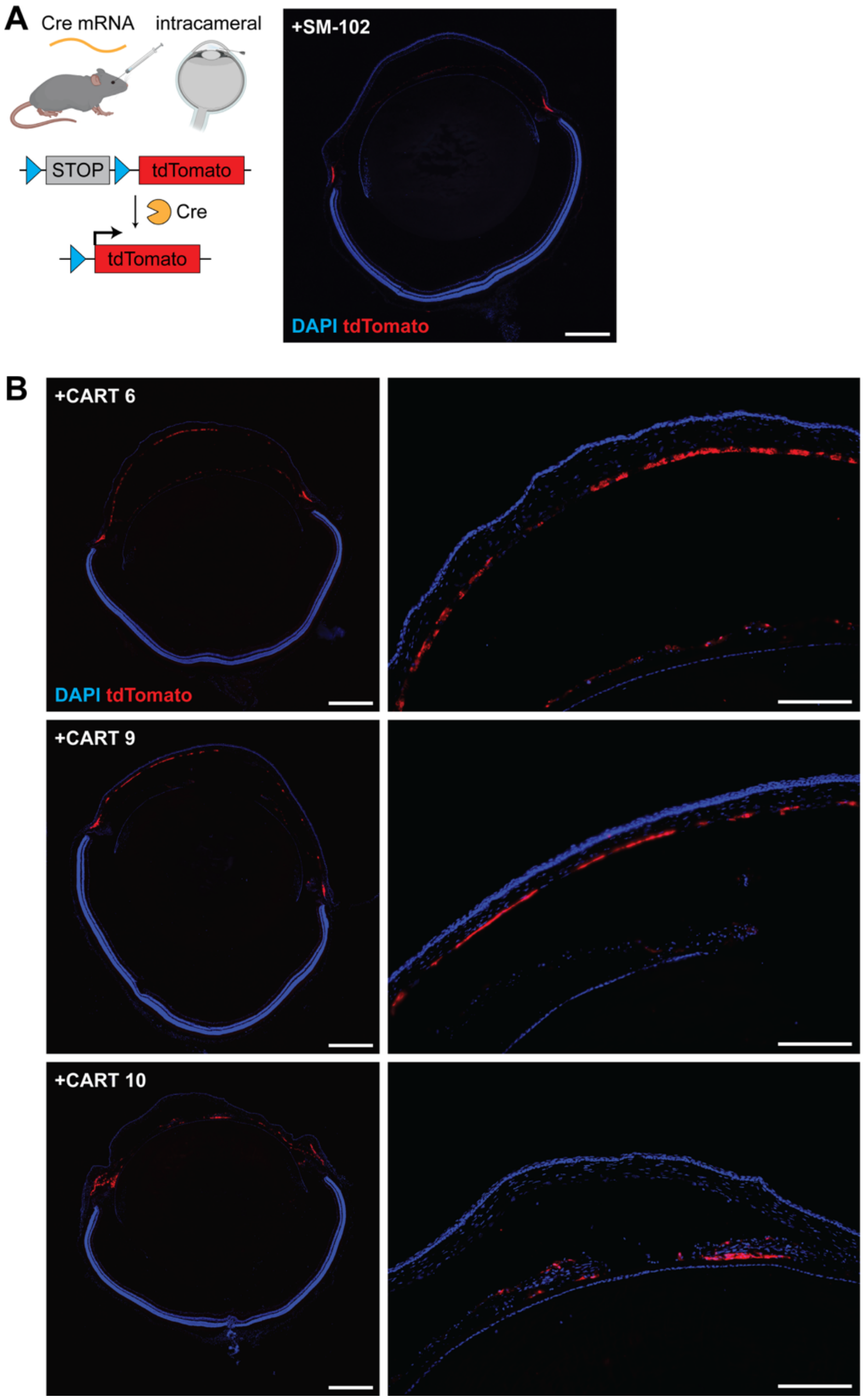

Figure S4. Intracameral administration of SM-102 LNP and guanidinylated serinol CARTs.

(A) Whole eye section from an Ai9 mouse 1 week after intracameral injection of Cre mRNA with SM-102 LNP. Eyes were dosed with 300 ng of RNA at a 6:1 charge ratio. n = 3 eyes. Scale bar, 500 µm.

(**B**) Whole eye and anterior segment sections from Ai9 mice 1 week after intracameral injection of Cre mRNA with CART 6, CART 9, or CART 10. Eyes were dosed with 300 ng of RNA at a 10:1 charge ratio. n = 3 eyes per group. Scale bars, 500 µm (left), 200 µm (right).

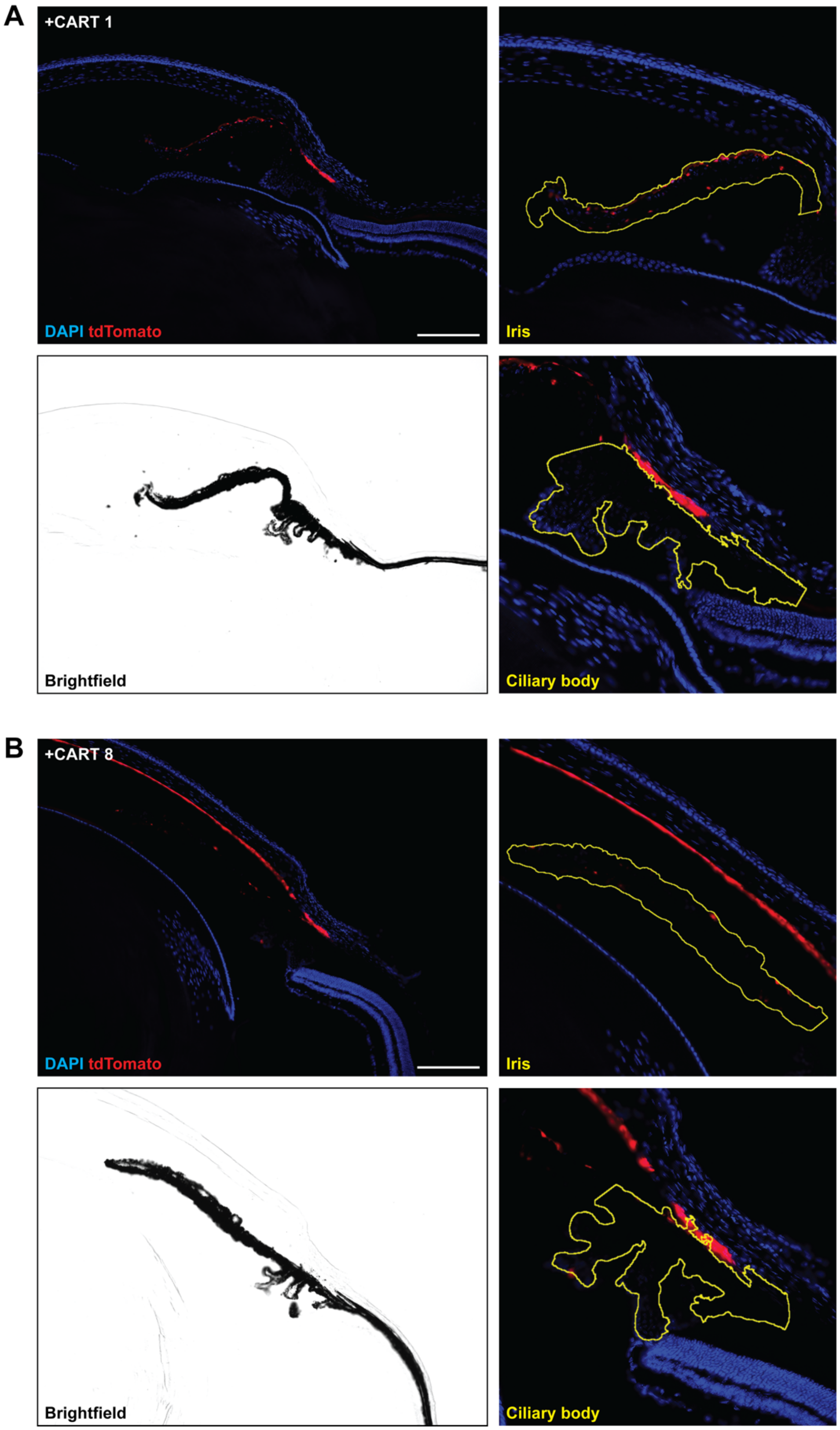

Figure S5. Transfection of the iridocorneal angle by intracameral CARTs.

(A,B) Iridocorneal angle sections from Ai9 mice 1 week after intracameral injection of Cre mRNA with CART 1 (A) or Cre mRNA with CART 8 (B). Eyes were dosed with 300 ng of RNA at a 10:1 charge ratio. Iris and ciliary body structures are outlined in yellow. n = 4 eyes per group. Scale bars, 200 μm.

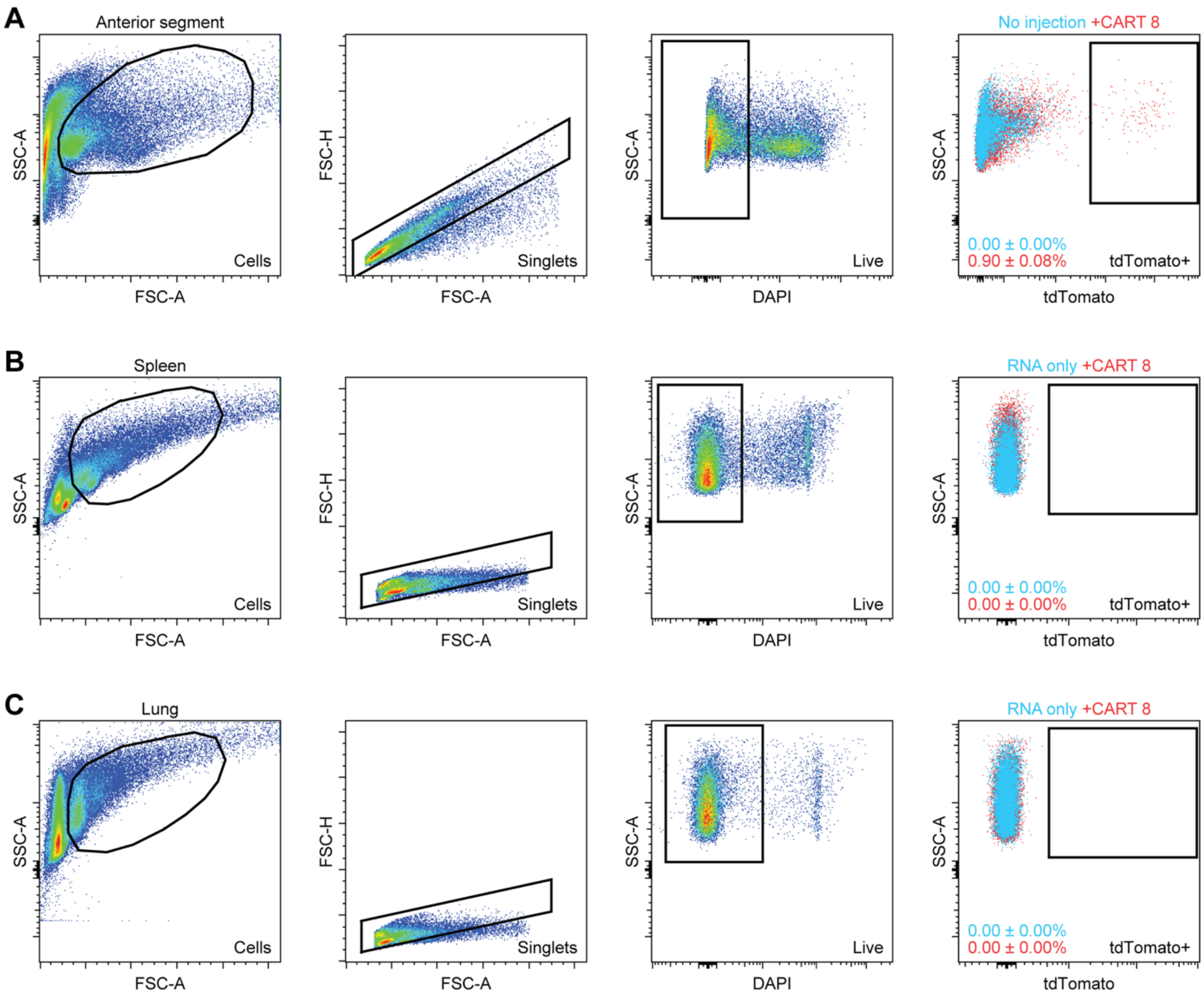

Figure S6. Organ tropism of intracameral CART 8.

(A) Representative FACS gating of anterior segments from Ai9 mice without treatment (blue) or 1 week after intracameral injection of Cre mRNA with CART 8 (red). Eyes were dosed with 300 ng of RNA at a 10:1 charge ratio. Numbers indicate mean ± SEM percent of tdTomato-positive cells among live cells. n = 3-6 eyes per group.

(B,C) Representative flow cytometry gating of spleens (B) and lungs (C) from Ai9 mice 1 week after intracameral injection of Cre mRNA only (blue) or Cre mRNA with CART 8 (red). Eyes were dosed with 300 ng of RNA at a 10:1 charge ratio. Numbers indicate mean ± SEM percent of tdTomato-positive cells among live cells. Mice were treated with the same condition in both eyes. n = 3 mice per group.

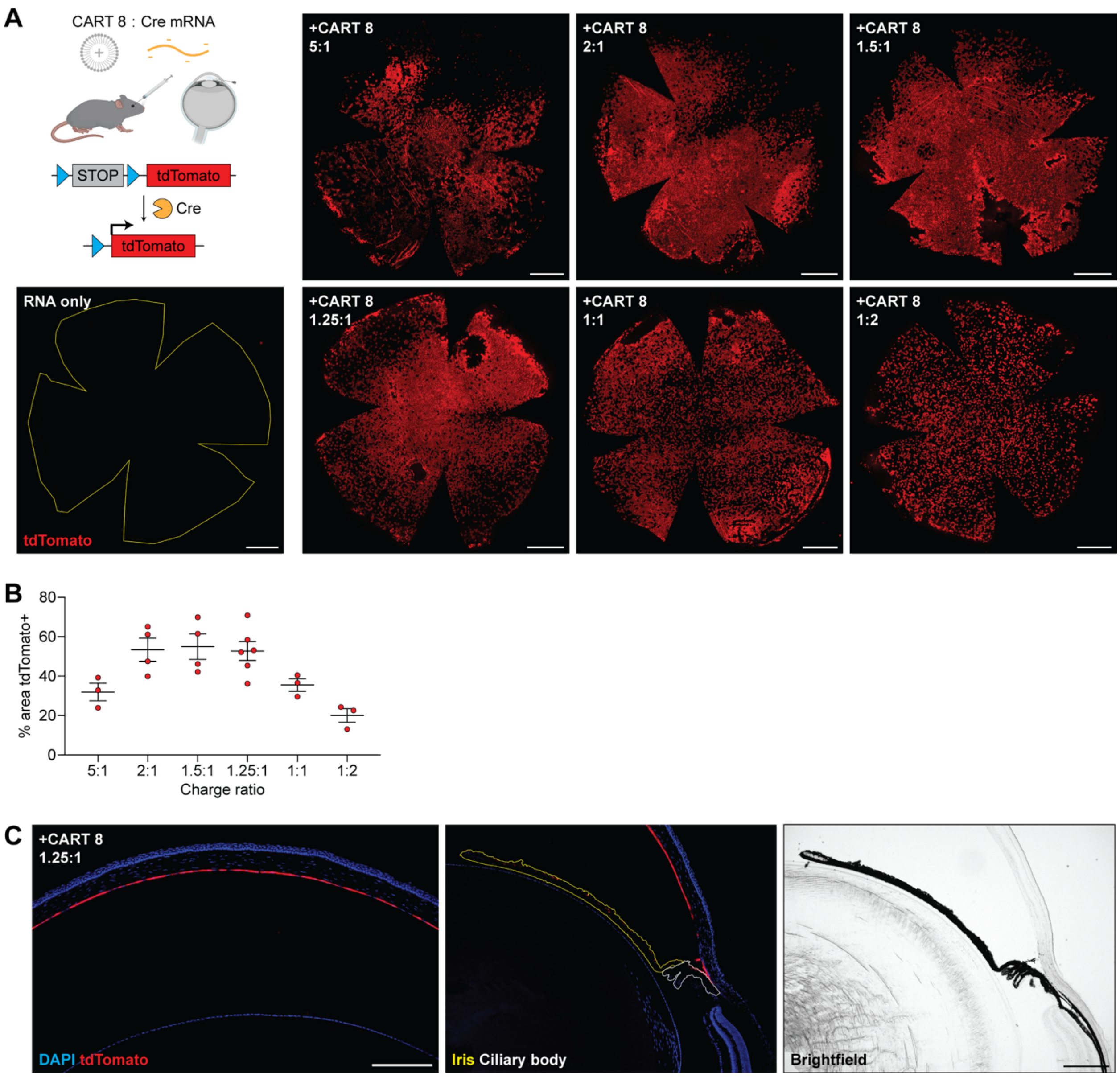

Figure S7. Effect of charge ratio on CART 8 transfection.

(A) Representative images of corneal flat mounts from Ai9 mice 1 week after intracameral injection of Cre mRNA only or Cre mRNA with CART 8. Eyes were dosed with 500 ng of RNA at the indicated charge ratios. n = 3-6 eyes per group. Scale bars, 500 µm.

(B) Quantification of tdTomato expression in Ai9 corneal flat mounts 1 week after intracameral injection of Cre mRNA with CART 8 at the indicated charge ratios. Eyes were dosed with 500 ng of RNA. Data are mean ± SEM. n = 3-6 eyes per group.

(C) Anterior segment and iridocorneal angle sections from an Ai9 mouse 1 week after intracameral injection of Cre mRNA with CART 8. Eyes were dosed with 500 ng of RNA at a 5:4 charge ratio. Iris and ciliary body structures are outlined in yellow and white, respectively. n = 4 eyes per group. Scale bars, 200 µm.

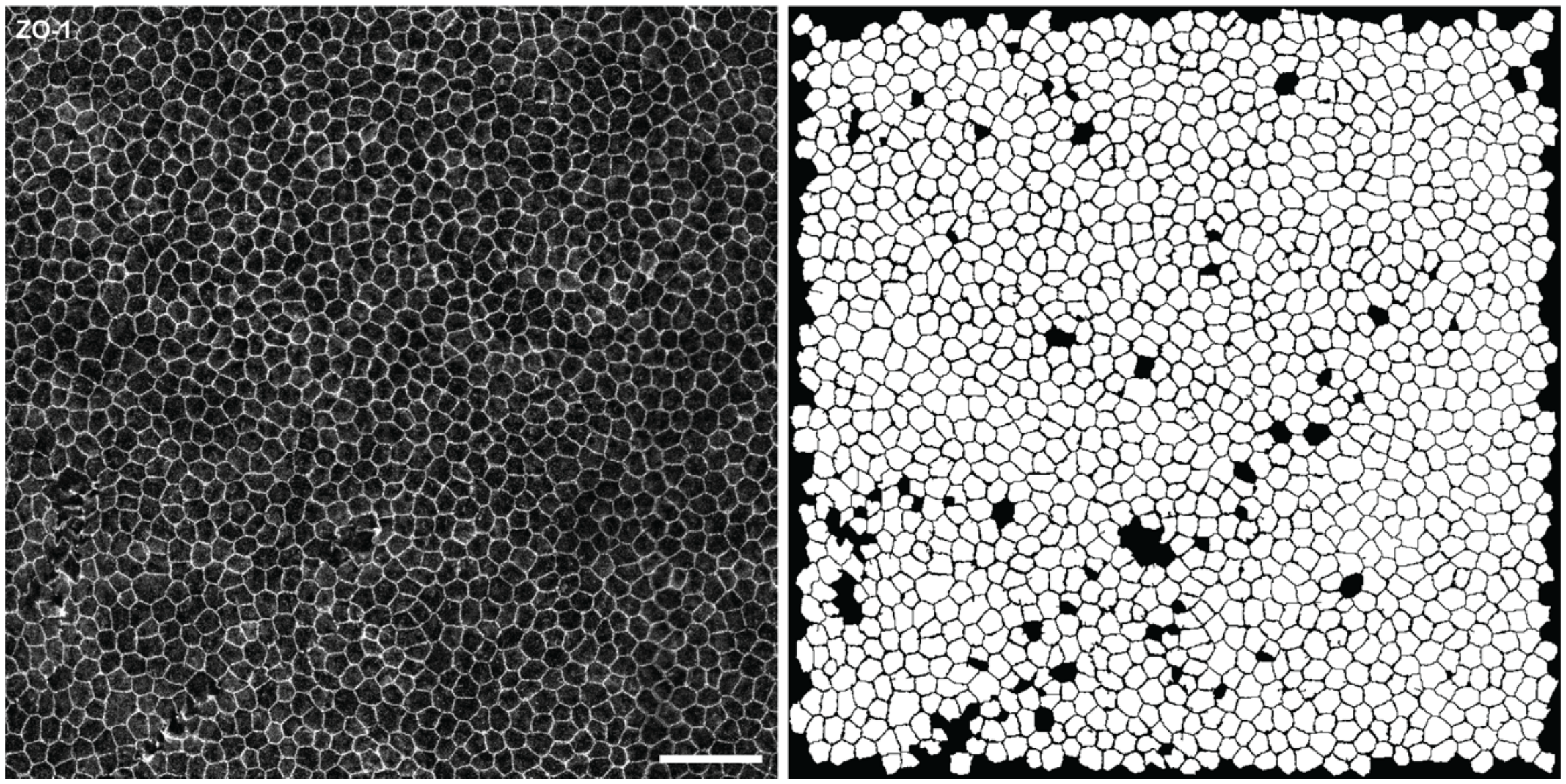

Figure S8. Automated segmentation of corneal endothelial cells.

Immunostaining for ZO-1 in a corneal flat mount (left) and the corresponding output following automated cell segmentation (right). Segmented cells are shown in white. Scale bars, 100 µm.

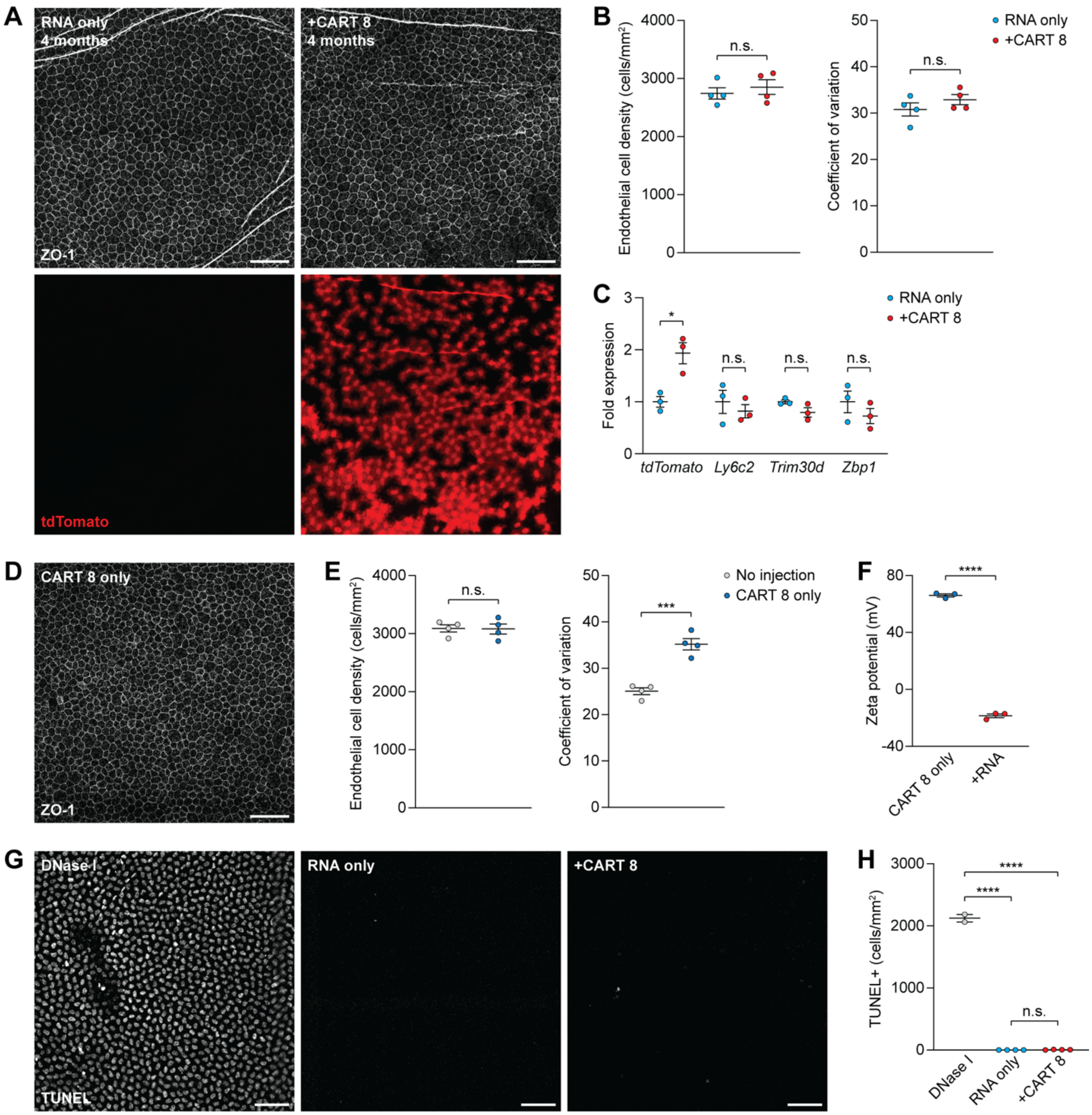

Figure S9. Safety of intracameral CART 8.

(A) Immunostaining for ZO-1 in corneal flat mounts from Ai9 mice 4 months after intracameral injection of Cre mRNA only or Cre mRNA with CART 8. Eyes were dosed with 500 ng of RNA at a 1.25:1 charge ratio. n = 4 eyes per group. Scale bars, 100 µm.

(B) Quantification of corneal endothelial cell density and coefficient of variation in anti-ZO-1-stained corneal flat mounts 4 months after intracameral injection of Cre mRNA only or Cre mRNA with CART 8. Data are mean ± SEM. n = 4 eyes per group. n.s., not significant.

(**C**) mRNA expression of indicated genes in anterior segments from Ai9 mice 4 months after intracameral injection of Cre mRNA only or Cre mRNA with CART 8. Data are mean ± SEM. n = 3 eyes per group. * $P<0.05$ by two-tailed Student's t test. n.s., not significant.

(D) Immunostaining for ZO-1 in a corneal flat mount from a C57BL/6J mouse 1 week after intracameral injection of CART 8 without RNA. Eyes were dosed with 1.25 µg of CART 8. n = 4 eyes. Scale bar, 100 µm.

(E) Quantification of corneal endothelial cell density and coefficient of variation in anti-ZO-1-stained corneal flat mounts 1 week after intracameral injection of CART 8 without RNA. Data for the no injection group are taken from Fig. 3, D and E. Data are mean ± SEM. n = 4 eyes per group. *** $P<0.001$ by two-tailed Student's t test. n.s., not significant.

(F) Surface charge of CART 8 with or without 1 µg of Cre mRNA at a 1.25:1 charge ratio in acidified 1x PBS (pH 5.5). Data are mean ± SEM. n = 3 technical replicates per group. **** $P<0.0001$ by two-tailed Student's t test.

(G) Terminal deoxynucleotidyl transferase dUTP nick end labeling (TUNEL) staining in corneal flat mounts from BALB/c mice treated with DNase I (positive control) or 48 hours after intracameral injection of luciferase mRNA only or luciferase mRNA with CART 8. Eyes were dosed with 500 ng of RNA at a 1.25:1 charge ratio. n = 2-4 eyes per group. Scale bars, 100 µm.

(H) Quantification of TUNEL staining in corneal flat mounts. Data are mean ± SEM. n = 2-4 eyes per group. **** $P<0.0001$ by two-tailed Student's t test. n.s., not significant.

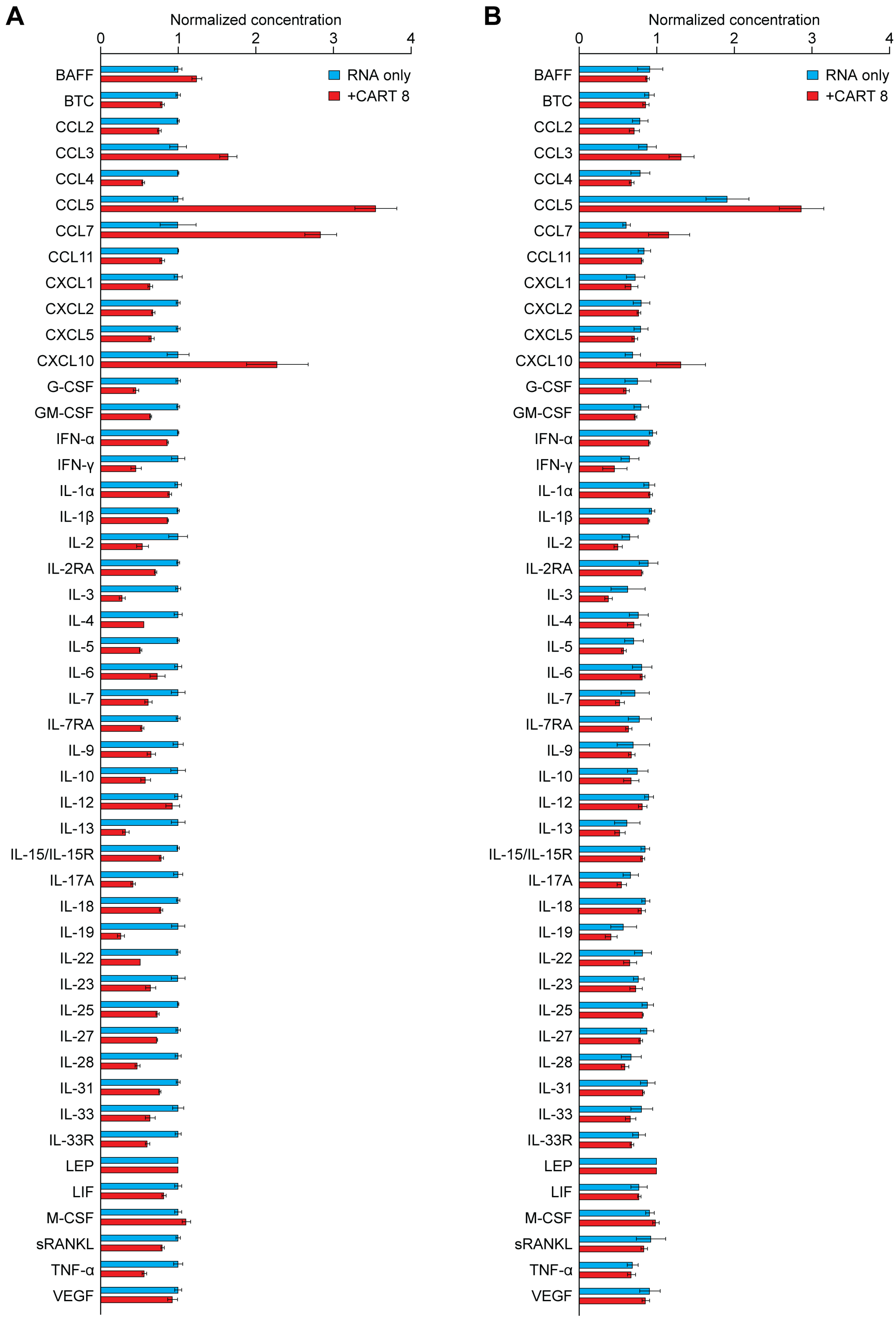

Figure S10. Cytokine levels after intracameral administration of CART 8.

(A,B) Cytokine levels in anterior segments from C57BL/6J mice 48 hours after intracameral dosing (A) or redosing (B) of EGFP mRNA with or without CART 8. Concentrations were normalized to mean values following initial treatment with EGFP mRNA only. Eyes were dosed with 500 ng of RNA at a 1.25:1 charge ratio. Redosing was performed after 1 week. Data are mean ± SEM. n = 4 eyes per group.

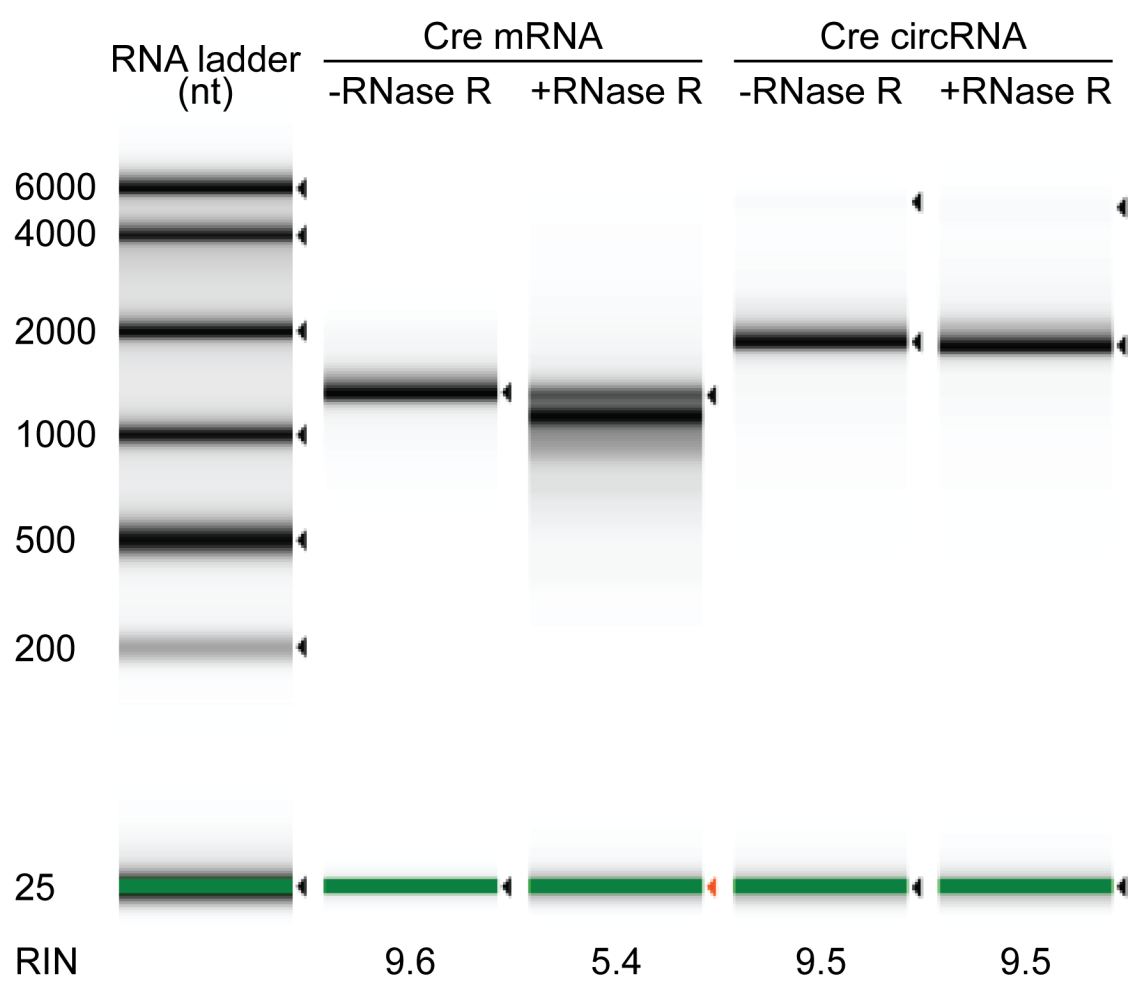

Figure S11. Validation of RNA circularity by RNase R.

TapeStation gel electrophoresis depicting the effect of RNase R digestion on Cre mRNA and circRNA. 10 µg of each RNA was incubated with 0.5 µL of RNase R in a 50 µL volume for 10 minutes at 37 °C. Data are from one of two experiments with similar results. RIN, RNA integrity number.

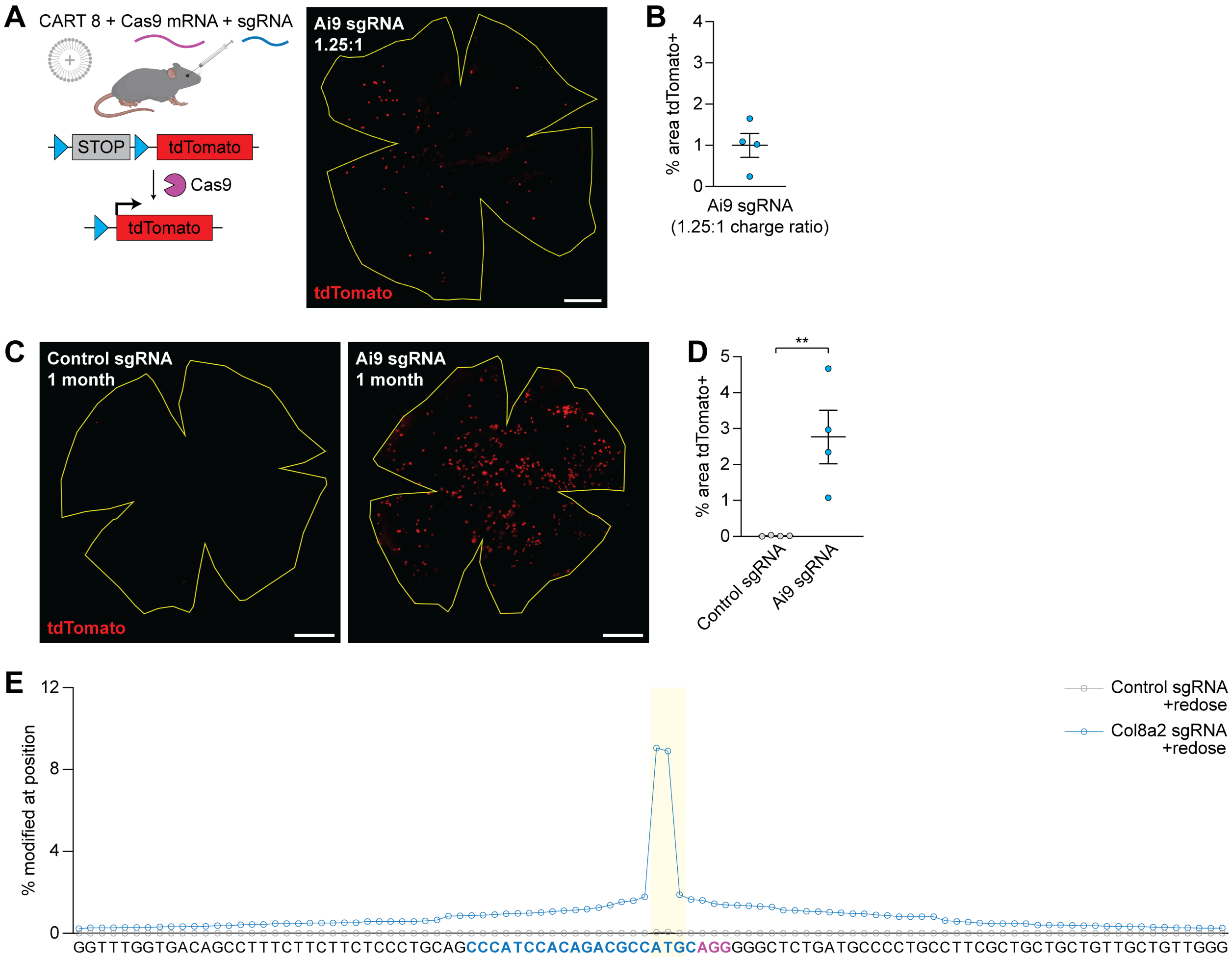

Figure S12. Quantification of CRISPR/Cas9 editing.

(A,B) Representative image (A) and quantification (B) of CRISPR/Cas9-mediated gene editing in Ai9 corneal flat mounts 1 week after intracameral injection of Cas9 mRNA and Ai9 sgRNA with CART 8. Eyes were dosed with 600 ng of total RNA at a 1.25:1 charge ratio. Data are mean ± SEM. n = 4 eyes. Scale bar, 500 µm.

(C,D) Representative images (C) and quantification (D) of CRISPR/Cas9-mediated gene editing in Ai9 corneal flat mounts 1 month after intracameral injection of Cas9 mRNA and the indicated sgRNA with CART 8. Eyes were dosed with 600 ng of total RNA at a 5:1 charge ratio. Data are mean ± SEM. n = 4 eyes per group. Scale bars, 500 µm. ** *P*<0.01 by two-tailed Student's t test.

(E) Editing activity at each nucleotide at the *Col8a2* locus 1 week after intracameral redosing of Cas9 mRNA and the indicated sgRNA with CART 8. Eyes were dosed with 600 ng of total RNA at a 5:1 charge ratio. Redosing was performed after 1 week. The Col8a2 sgRNA sequence is depicted in blue. The protospacer adjacent motif (PAM) sequence is depicted in purple. The *Col8a2* start codon is highlighted in yellow. n = 4-6 eyes per group.

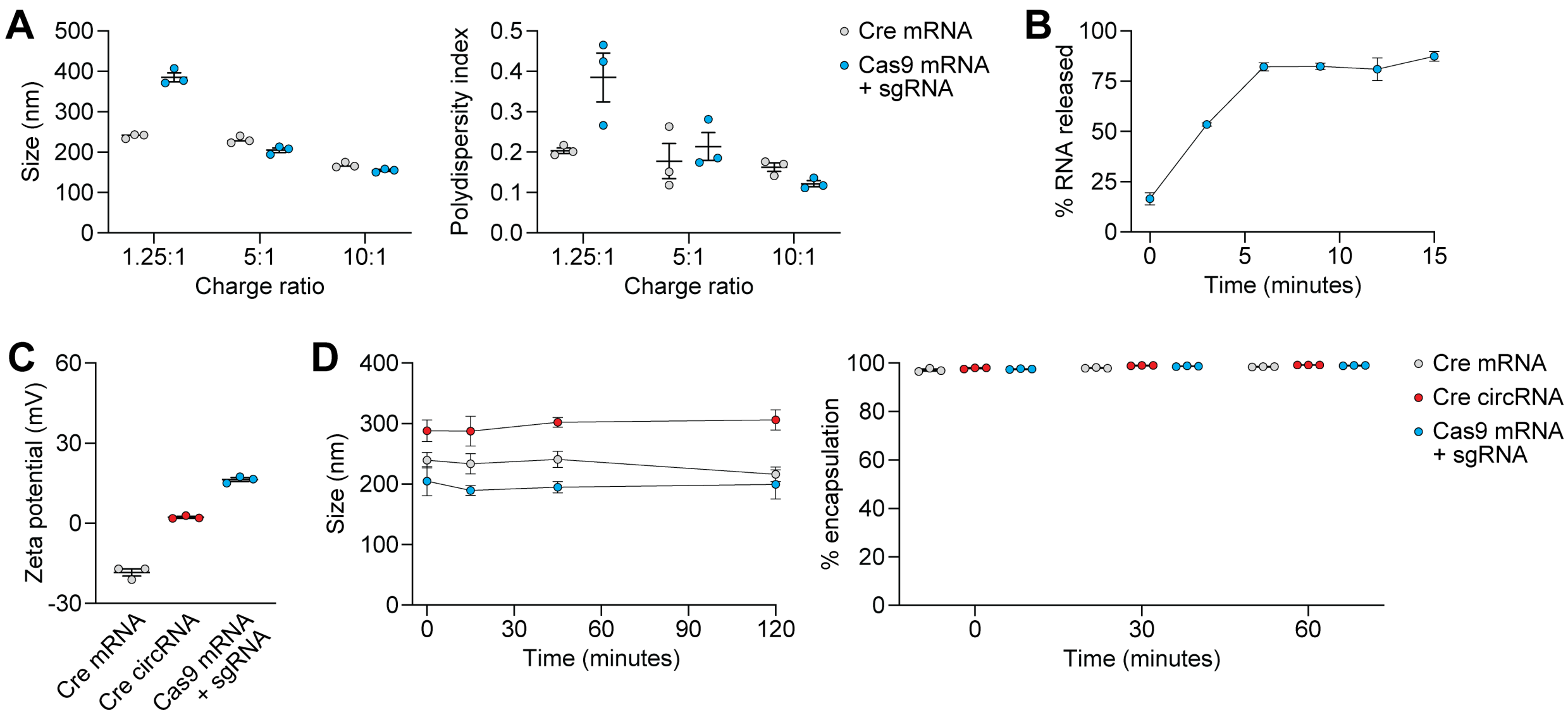

Figure S13. Effect of cargo on CART-RNA formulations.

(A) Particle size and polydispersity index of CART 8 complexed with 1 µg of Cre mRNA or Cas9 mRNA and Ai9 sgRNA at the indicated charge ratios in acidified 1x PBS. Data are mean ± SEM. n = 3 technical replicates per group.

(B) RNA release profiles at the indicated time points after formulation of CART 8 with 1 µg of Cas9 mRNA and Ai9 sgRNA at a 5:1 charge ratio in acidified 1x PBS. Na_2CO_3 was added at time = 0. Data are mean ± SEM. n = 3 technical replicates per group.

(C) Surface charge of CART 8 complexed with 1 µg of Cre mRNA (1.25:1 charge ratio), Cre circRNA (1.25:1 charge ratio), or Cas9 mRNA and Ai9 sgRNA (5:1 charge ratio) in acidified 1x PBS. Data are mean ± SEM. Values for Cre mRNA are the same as in fig. S9F. n = 3 technical replicates per group.

(D) Size and encapsulation rate at the indicated time points after formulation of CART 8 with 1 µg of Cre mRNA (1.25:1 charge ratio), Cre circRNA (1.25:1 charge ratio), or Cas9 mRNA and Ai9 sgRNA (5:1 charge ratio) in acidified 1x PBS. Data are mean ± 95% CI for size, mean ± SEM for encapsulation rate. n = 3 technical replicates per group.

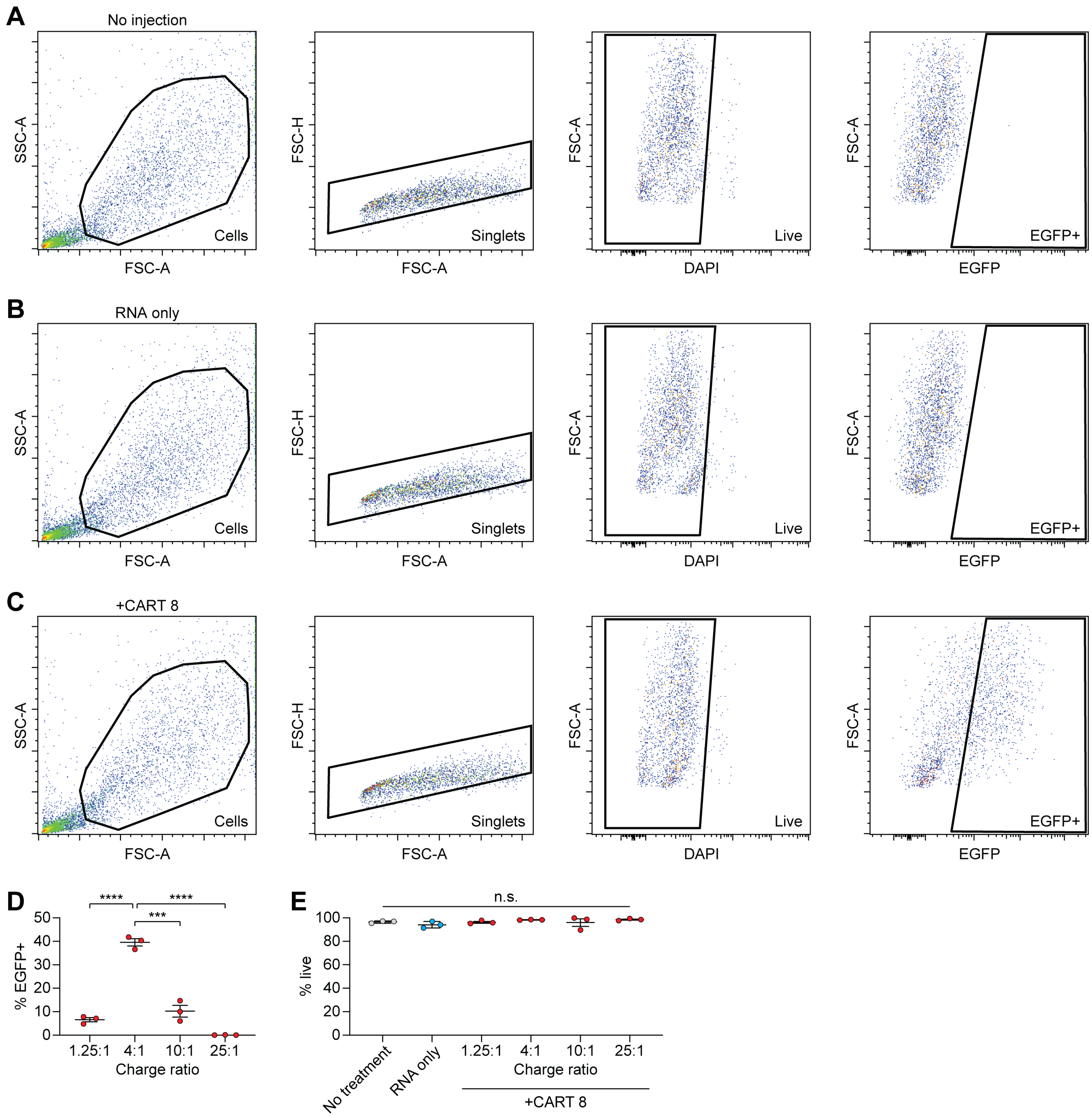

Figure S14. Transfection of primary human corneal endothelial cells.

(A-C) Representative flow cytometry gating of primary human corneal endothelial cells without treatment (A) or 24 hours after treatment with EGFP mRNA only (B) or EGFP mRNA with CART 8 (C). Cells were treated with 100 ng of RNA per 96-well at a 4:1 charge ratio. n = 3 biological replicates per group.

(D) Flow cytometry quantification of EGFP expression in HCECs 16 hours after treatment with EGFP mRNA with CART 8 at the indicated charge ratios. Cells were treated with 100 ng of RNA per 96-well. Data are mean ± SEM. Values for 4:1 are the same as in Fig. 5B. n = 3 biological replicates per group. *** P<0.001, **** P<0.0001 by two-tailed Student's t test.

(E) Flow cytometry quantification of HCEC viability without treatment or 16 hours after treatment with EGFP mRNA only or EGFP mRNA with CART 8 at the indicated charge ratios. Cells were treated with 100 ng of RNA per 96-well. Data are mean ± SEM. n = 3 biological replicates per group. n.s., not significant.

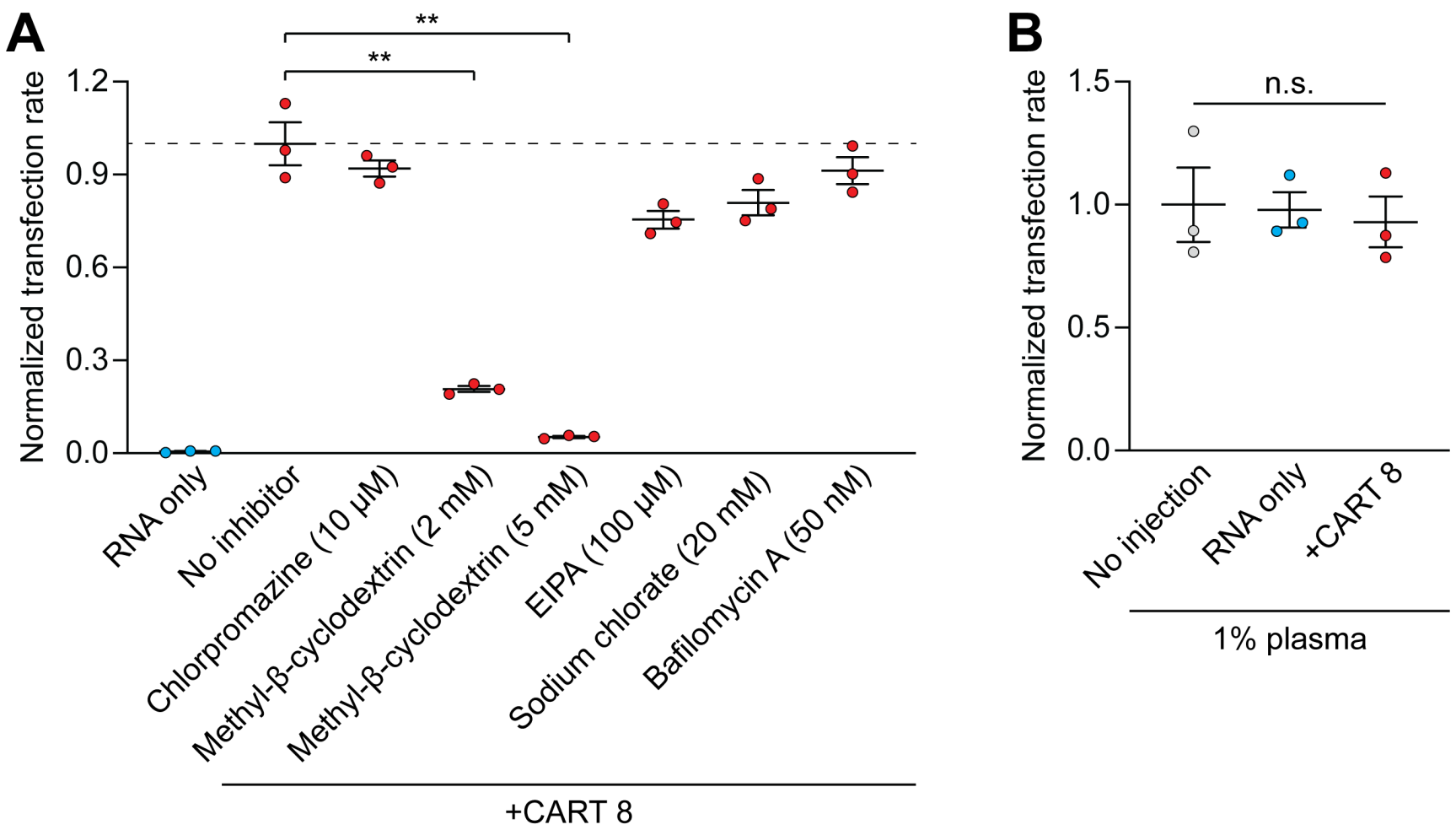

Figure S15. Factors affecting in vitro CART 8 transfection.

(A) Flow cytometry quantification of EGFP expression in HCECs 16 hours after treatment with EGFP mRNA with CART 8 in the presence of the indicated small molecule inhibitors. Transfection rates were normalized to the mean value in the absence of inhibitors (dashed line). Cells were treated with 100 ng of RNA per 96-well at a 4:1 charge ratio. Data are mean ± SEM. n = 3 biological replicates per group. ** $P<0.01$ by two-tailed Student's t test with Bonferroni correction.

(B) Flow cytometry quantification of EGFP expression in HCECs 16 hours after treatment with EGFP mRNA with CART 8 in the presence of plasma from untreated or treated mice. Transfection rates were normalized to the mean value in the presence of plasma from untreated mice. Plasma from treated mice was collected 9 days after intracameral dosing and 48 hours after redosing of 500 ng of EGFP mRNA with or without CART 8. Cells were treated with 100 ng of RNA per 96-well at a 4:1 charge ratio. Data are mean ± SEM. n = 3 biological replicates per group. n.s., not significant.

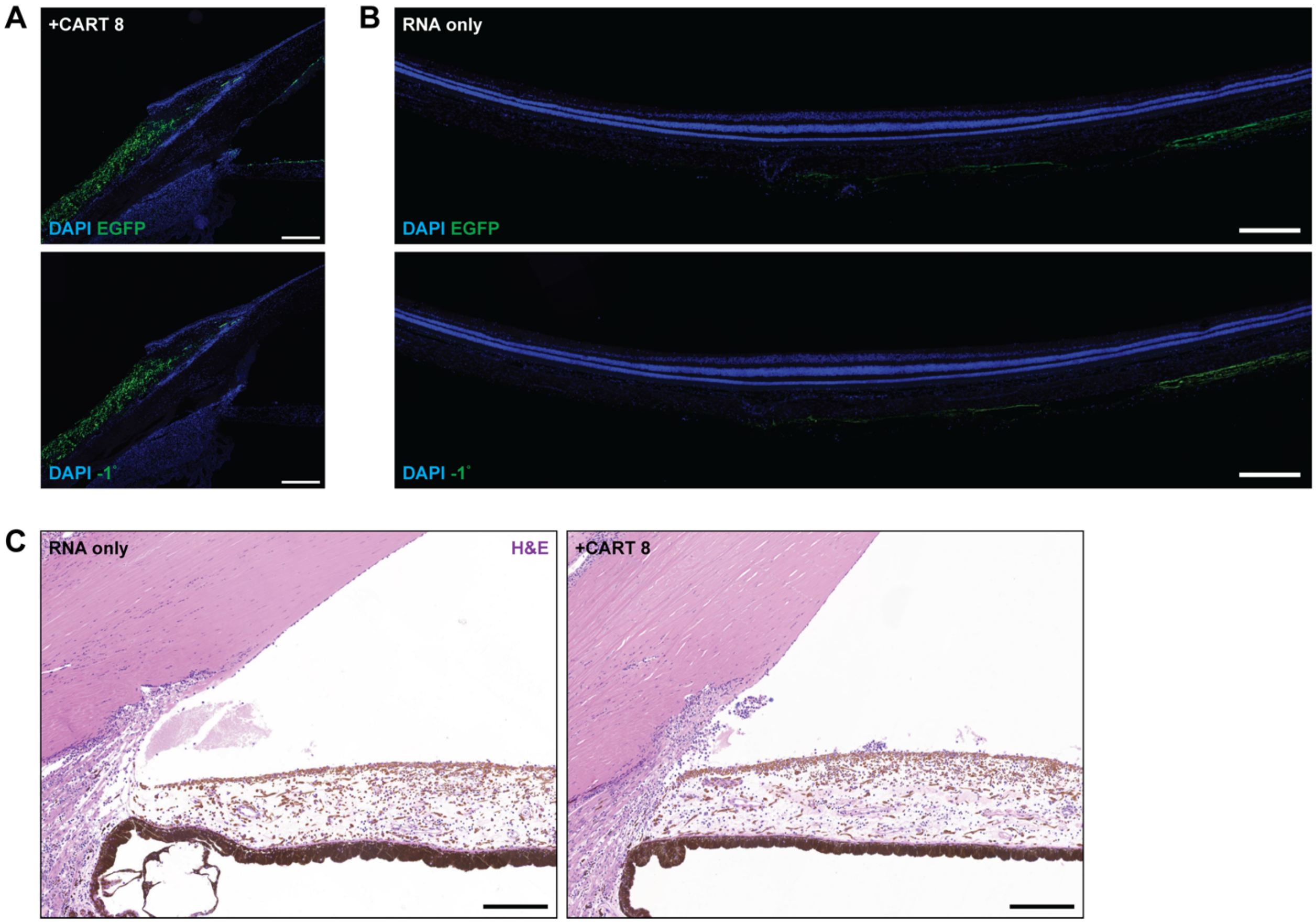

Figure S16. Additional NHP histology.

(A,B) Immunostaining for EGFP at the limbus (A) and posterior wall (B) of NHP eyes in the presence (top) and absence (bottom) of primary anti-GFP antibody. Top image in (B) is the same as in Fig. 5G. n = 1 eye per group. Scale bars, 100 µm (A), 500 µm (B).

(C) Hematoxylin and eosin (H&E) staining of the iridocorneal angle of NHP eyes 48 hours after intracameral injection of EGFP mRNA only or EGFP mRNA with CART 8. n = 1 eye per group. Scale bars, 200 µm**.**

Table S1. Primer sequences.

Gene	5’	3’
Gapdh	AGGTCGGTGTGAACGGATTTG	TGTAGACCATGTAGTTGAGGTCA
tdTomato	ATCGTGGAACAGTACGAGCG	TGAACTCTTTGATGACGGCCA
Ly6c2	GCGCCTCTGATGGATTCTGCAT	TCCTTGATTGGCACACCAGCAG
Trim30d	TATGTCCTGACAAGCATTCAGAC	GCCCTTCAAATGACTGTAGCA
Zbp1	AAGAGTCCCCTGCGATTATTTG	TCTGGATGGCGTTTGAATTGG
Col8a2 (outer)	GAAGGGTTCTGTCTGGTCGC	TACGGTAGAGTAGGGGAGGC
Col8a2 (inner)	GGAGACTGGTCAGGTCTGGA	CAAGTACTGGCCTTTGCCCT

Data S1. (separate file)

RNA-seq of tdTomato-positive cells after intracameral injection of Cre mRNA with CART 8 compared to cells from untreated anterior segments.

Data S2. (separate file)

RNA-seq of anterior segments after intracameral injection of EGFP mRNA with CART 8 compared to EGFP mRNA only.
